# A p53-Dependent Checkpoint Induced upon DNA Damage Alters Cell Fate during hiPSC Differentiation

**DOI:** 10.1016/j.stemcr.2020.08.003

**Published:** 2020-09-03

**Authors:** Cara B. Eldridge, Finian J. Allen, Alastair Crisp, Rodrigo A. Grandy, Ludovic Vallier, Julian E. Sale

**Affiliations:** 1 MRC Laboratory of Molecular Biology, Francis Crick Avenue, Cambridge CB2 0QH, UK; 2 Department of Chemistry, University of Cambridge, Cambridge CB2 1EW, UK; 3 Wellcome–MRC Cambridge Stem Cell Institute, Anne McLaren Laboratory, University of Cambridge, Cambridge CB2 0SZ, UK; 4 Department of Surgery, University of Cambridge, Cambridge CB2 0QQ, UK; 5 Wellcome Sanger Institute, Wellcome Genome Campus, Hinxton CB10 1SA, UK

**Keywords:** DNA-damage response, p53, differentiation, definitive endoderm, checkpoint, hiPSC, hESC

## Abstract

The ability of human induced pluripotent stem cells (hiPSCs) to differentiate *in vitro* to each of the three germ layer lineages has made them an important model of early human development and a tool for tissue engineering. However, the factors that disturb the intricate transcriptional choreography of differentiation remain incompletely understood. Here, we uncover a critical time window during which DNA damage significantly reduces the efficiency and fidelity with which hiPSCs differentiate to definitive endoderm. DNA damage prevents the normal reduction of p53 levels as cells pass through the epithelial-to-mesenchymal transition, diverting the transcriptional program toward mesoderm without induction of an apoptotic response. In contrast, *TP53*-deficient cells differentiate to endoderm with high efficiency after DNA damage, suggesting that p53 enforces a “differentiation checkpoint” in early endoderm differentiation that alters cell fate in response to DNA damage.

## Highlights

• DNA damage impairs the efficiency and fidelity of human stem cell differentiation
• p53 is reduced transiently during the commitment to definitive endoderm
• Preventing p53 reduction diverts cells away from endoderm without apoptosis
• p53-deficient cells differentiate to endoderm efficiently in the face of DNA damage

## Teaser

Eldridge et al. demonstrate a temporal window during which the differentiation of human iPSCs to definitive endoderm is sensitive to low levels of DNA damage, causing diversion of the differentiation program toward a more mesodermal fate. This effect is almost entirely dependent on p53, with both the efficiency and the fidelity of endoderm differentiation being restored in damaged p53-deficient cells.

## Introduction

*In vitro* differentiation of human induced pluripotent stem cells (hiPSCs) and embryonic stem cells (hESCs) to definitive endoderm (DE) is induced by activation of the Activin/Nodal, FGF, BMP4, and WNT signaling pathways in concert with inhibition of phosphoinositide 3-kinases (Vallier et al., 2009). Downregulation of the core pluripotency transcription factors *SOX2*, *NANOG*, and *POU5F1* increases expression of *EOMES* (Teo et al., 2011), which drives a transition through a primitive streak-like stage (Arnold et al., 2008). The cells then undergo the epithelial-to-mesenchymal transition (EMT) and acquire markers of DE, including *SOX17* (Kanai-Azuma et al., 2002) and *FOXA2* (Dufort et al., 1998).

p53 is central to the cellular response to DNA damage (Williams and Schumacher, 2016). In somatic cells, p53 is maintained at a low level through degradation induced by the E3 ubiquitin ligase MDM2 (Michael and Oren, 2003). In response to DNA damage, a well-described signaling cascade leads to ATM-dependent phosphorylation of p53 and its stabilization (Banin et al., 1998; Canman et al., 1998). Activated p53 then drives a transcriptional program leading to cell-cycle arrest to facilitate DNA repair and, if unsuccessful, senescence or apoptosis (Shiloh and Ziv, 2013).

In mouse embryonic stem cells p53 activation can lead to differentiation through suppression of the pluripotency factor *Nanog* (Lin et al., 2005) and modulation of WNT signaling (Lee et al., 2010). This leads to damaged cells being removed from the stem cell pool, limiting their capacity to propagate genetic defects (Lin et al., 2005). Nonetheless, *Trp53*-null mice largely develop normally (Donehower et al., 1992), although female mice are highly susceptible to exencephaly (Armstrong et al., 1995; Sah et al., 1995). In contrast, in *Xenopus* embryos depleted of p53, mesoderm differentiation is inhibited (Cordenonsi et al., 2003) and gastrulation is not completed (Wallingford et al., 1997). These differences may be explained at least in part by the availability of other p53 family members in mammalian cells, p63 and p73, which together with p53 are important for coordination of signaling pathways controlling mesendoderm differentiation (Wang et al., 2017) by inducing expression of genes, including members of the *Wnt* and *Fzd* families (Lee et al., 2010). WNT activates TCF3, which, with SMAD2/3, binds to enhancers of mesendoderm genes and activates their transcription (Wang et al., 2017).

Elevated p53 levels in the early embryo decline significantly during embryonic development (Schmid et al., 1991). Mice lacking one of the p53 regulators *Mdm2* or *Mdm4* do not survive embryonic development, and this can be rescued by concurrent disruption of *Trp53* (Jones et al., 1995; Montes de Oca Luna et al., 1995; Parant et al., 2001). Conversely, overexpression of *Trp53* perturbs renal differentiation, further suggesting that tight control of p53 is required for successful development (Godley et al., 1996). Despite this work on the roles of p53 in both differentiation and the response of stem cells to DNA damage, surprisingly little is known about the p53-dependent response to DNA damage in a differentiating stem cell. Here we address this question using an iPSC model of human endoderm differentiation.

## Results

### DNA Damage Decreases the Efficiency of hiPSC Differentiation to DE

**Figure 1:**
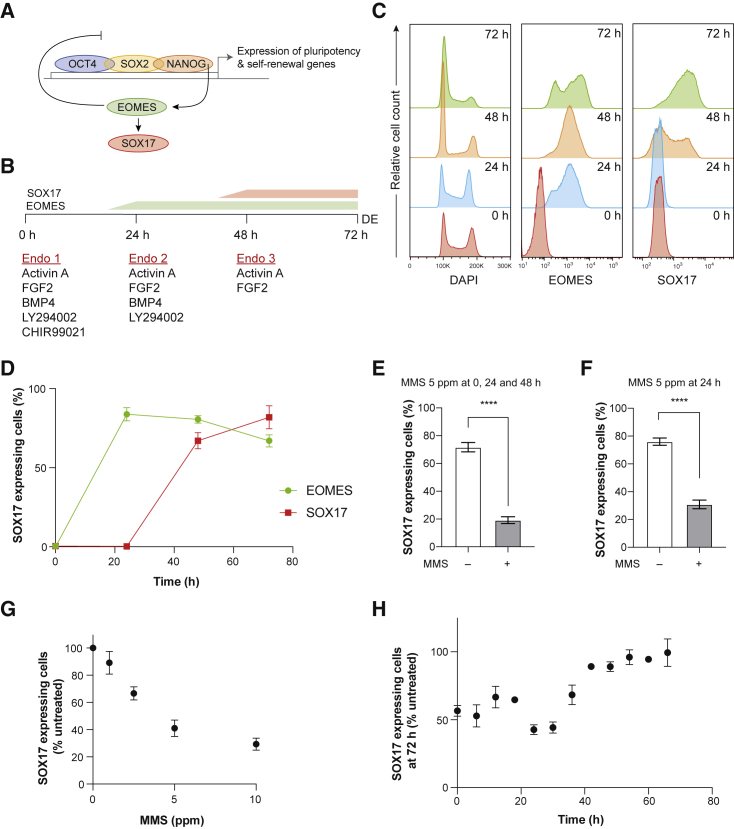
DNA Damage Inhibits Endoderm Differentiation in hiPSCs (A) Highly simplified genetic circuit controlling differentiation to DE. (B) Scheme for *in vitro* endoderm differentiation over 72 h. (C) Permeabilized flow cytometry to monitor expression of EOMES (center) and SOX17 (right) during differentiation to DE. DNA content (DAPI, left) shows the change in the cell-cycle profile during differentiation, with a marked shift toward G1. (D) Kinetics of EOMES and SOX17 expression during DE differentiation (n = 4 indpendent experiments, mean ± SEM is plotted). (E) Inhibition of DE differentiation by exposure to MMS throughout differentiation. MMS (5 ppm) was administered at 0, 24, and 48 h (n = 8 independent experiments, ^∗∗∗∗^p < 0.0001 using a paired t test, mean ± SEM is plotted). (F) Inhibition of DE differentiation by exposure to a single dose of 5 ppm MMS at 24 h (n = 12 independent experiments, ^∗∗∗∗^p < 0.0001 using a paired t test, mean ± SEM is plotted). (G) Dose-dependent inhibition of DE differentiation by MMS treatment at 24 h (n = 5 independent experiments, mean ± SEM is plotted). (H) Cells were treated with 5 ppm MMS every 6 h during differentiation and the efficiency of differentiation at 72 h was monitored (n ≥ 2 independent experiments for each time point, mean ± SEM is plotted).

We employed the BOBSC hiPSC line (Andersson-Rolf et al., 2017), a derivative of cA1ATD (Yusa et al., 2011), and an established protocol for driving DE differentiation over 72 h (Yiangou et al., 2019) (Figures 1A and 1B). Differentiation was initially monitored using flow cytometry on permeabilized cells with antibodies against the mesendoderm marker EOMES and against SOX17, which marks DE (Arnold et al., 2008; Kanai-Azuma et al., 2002). The proportion of cells expressing SOX17 at 72 h thus provides a simple assay to monitor the efficiency of differentiation (Figures 1C and 1D). Detection of SOX17 by flow cytometry closely mirrored changes in mRNA levels during differentiation (Figure S1A).

To assess the impact of DNA damage on endoderm differentiation, we treated cells with 5 ppm methyl methanesulfonate (MMS) at 0, 24, and 48 h after initiating differentiation (Figure 1B). MMS generates 7-methylguanine and 3-methyladenine in DNA (Beranek, 1990), which can lead to replication stalling and DNA damage (Lundin et al., 2005). It has a half-life of about 4.5 h in aqueous solution (PubChem: CID 4156 https://pubchem.ncbi.nlm.nih.gov/compound/4156). MMS treatment resulted in a significant reduction in SOX17-expressing cells at 72 h (Figure 1E). A single dose of MMS at 24 h (Figure 1B) had a similar effect (Figure 1F) and did not alter the number of cell divisions (Figure S1B) or cell viability (Figure S1C). Likewise, a single low dose of UV-C (2 J/m^2^) at 24 h reduced the proportion of SOX17-expressing cells at 72 h (Figure S1D). Furthermore, the decrease in efficiency of differentiation was proportional to the dose of MMS (Figure 1G) and UV (Figure S1E). Importantly, the reduction in SOX17-positive cells at 72 h following damage was not simply the result of differentiation delay, as continuing to 96 h did not increase the proportion of cells expressing SOX17 (Figure S1F).

In order to ensure that the damage-induced decrease in the efficiency of differentiation was not a specific feature of the BOBSC hiPSCs, we also assessed differentiation of the H9 hESC line (Thomson et al., 1998), which had a differentiation efficiency similar to that of BOBSC (Figure S1G). Treatment with both MMS and UV irradiation at 24 h likewise significantly decreased the number of cells expressing SOX17 at 72 h (Figures S1H and S1I), confirming that the effect of DNA damage on differentiation is likely to be generalizable.

We next determined the effect of DNA damage on differentiation efficiency as a function of time of exposure during the protocol. A single dose of 5 ppm MMS was administered at different times during the differentiation protocol and the proportion of cells expressing SOX17 at 72 h measured (Figure 1H). MMS had a maximal inhibitory effect on differentiation when administered in a window between ~20 and 34 h. Treatment after 40 h had very little effect on the outcome of differentiation, when a proportion of cells had begun expressing SOX17. This suggested that once the cells have committed to DE, the outcome cannot be altered by DNA damage.

### A Fall in p53 Level during Early Endoderm Differentiation Is Prevented by DNA Damage

**Figure 2:**
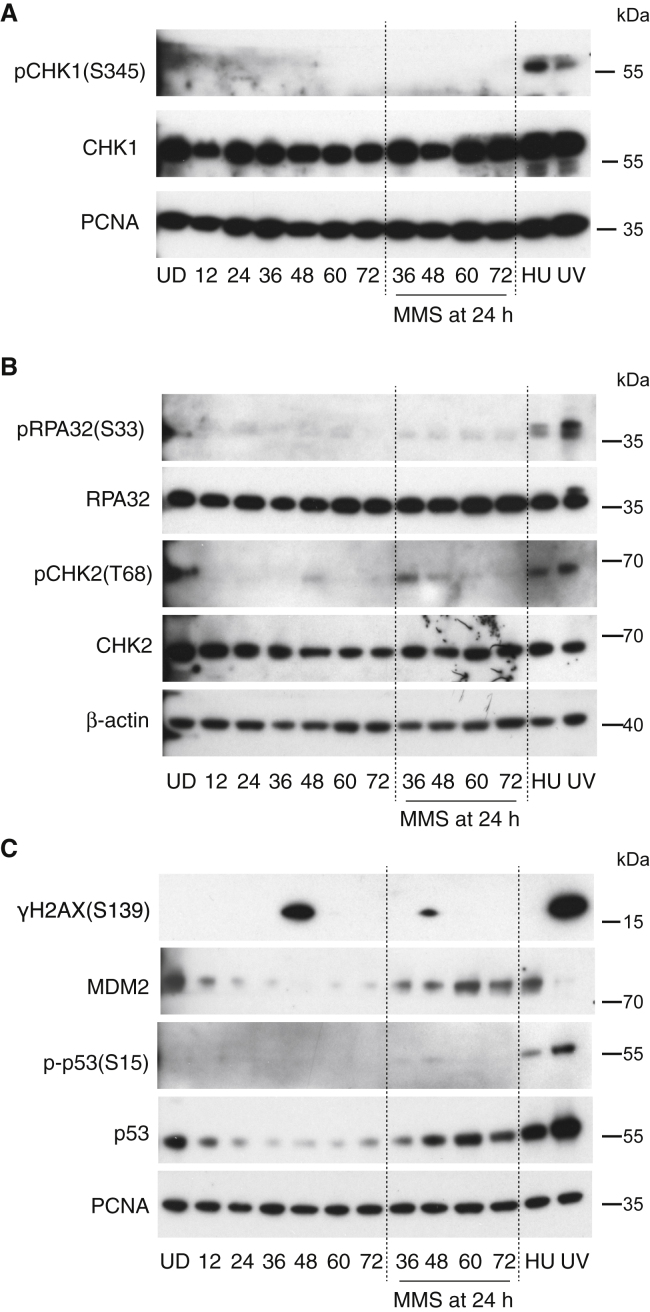
DNA Damage during Early Endoderm Differentiation Prevents Reduction of p53 Levels Western blot analyses of changes to the DNA-damage response during differentiation, with and without 5 ppm MMS treatment at 24 h. Protein was collected from whole-cell lysates every 12 h. Positive control samples were collected for protein 5 h after treatment, including 2 mM HU and 20 J/m^2^ UV-C. (A) Phospho-CHK1 (Ser-345) and total CHK1, with PCNA as a loading control. (B) Phosphorylation of RPA32 (Ser-33), total RPA, phosphorylation of CHK2 (Thr-68), total CHK2, and β-ACTIN as a loading control. (C) Phospho-H2AX (Ser-139), total MDM2, phospho-p53 (Ser-15), and total p53. PCNA is used as a loading control.

To understand the role of DNA-damage-response (DDR) pathways throughout differentiation, we monitored key features of the DDR by western blotting (Figure 2). The total levels of key DDR kinases CHK1 and CHK2, as well as one of their substrates, RPA, did not change during the differentiation protocol (Figures 2A and 2B). However, p53 levels fell as differentiation proceeded (Figure 2C), but were increased following treatment with 5 ppm MMS at 24 h (Figure 2C). Interestingly, while the low dose of MMS used in these experiments stabilized p53 and induced MDM2 expression, it was accompanied by minimal or no phosphorylation of the DNA-damage markers CHK1, CHK2, and RPA; H2AX; or p53 itself. A somewhat surprising observation in this context was a spike of phosphorylation of the histone variant H2AX around 50 h into differentiation (Figure S2). Phosphorylated H2AX (γH2AX) is frequently used as a surrogate marker for DNA double-strand breaks (Rogakou et al., 1998) and was therefore unexpected during normal differentiation. The observed spike of γH2AX was not induced by exogenous DNA damage; indeed, it was attenuated by it (Figure 2C) and was also not accompanied by significant DNA-damage signaling (Figure 2). We speculate that it may be related to the EMT through which the cells pass at this time (Teo et al., 2011). Indeed, H2AX phosphorylation during the EMT in cancer cell line models has previously been shown to play a role in transcriptional regulation of the vast gene expression changes that occur (Singh et al., 2015).

### Loss of p53 Rescues DE Differentiation after DNA Damage

**Figure 3:**
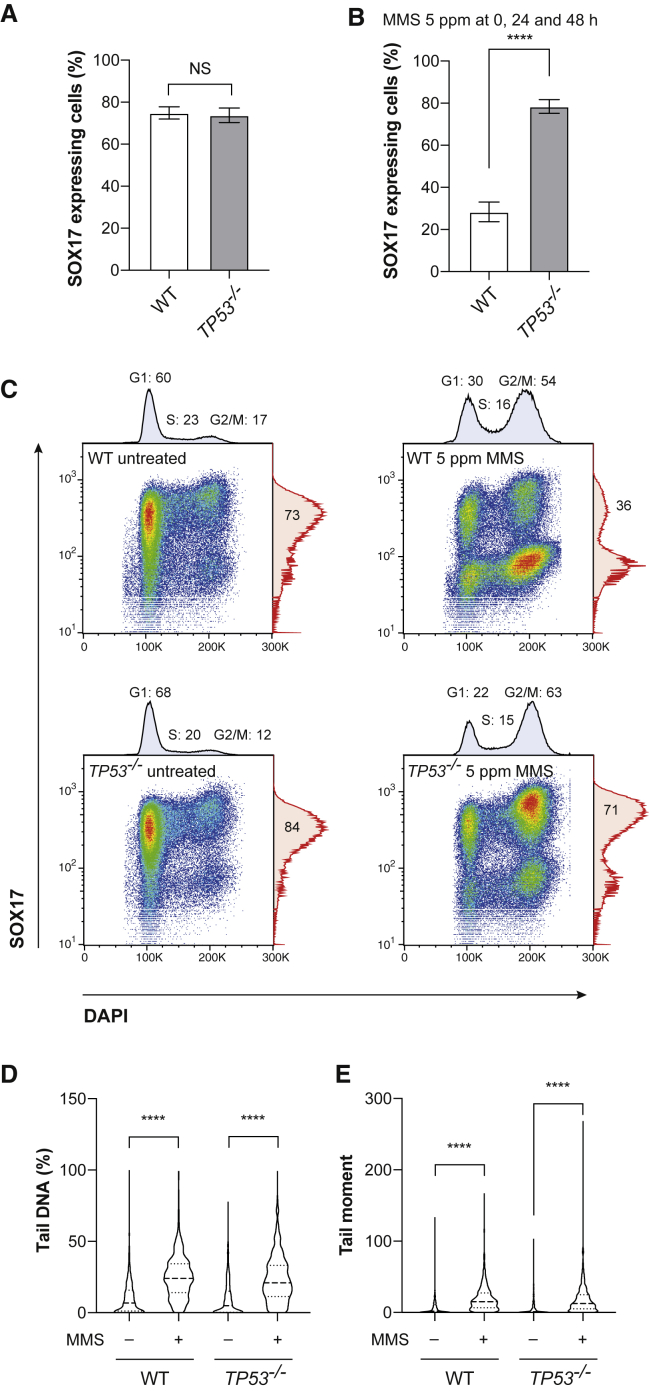
Loss of *TP53* Rescues DE Differentiation after DNA Damage (A) The percentage of cells expressing SOX17 at 72 h of differentiation in the wild-type (WT) and *TP53*^−/−^ cell line. No difference in the efficiency of differentiation was seen between the two cell lines using an unpaired t test (p = 0.8126, n = 6 independent experiments, mean ± SEM is plotted). (B) WT and *TP53*^−/−^ cell lines were treated with 5 ppm MMS at 0, 24, and 48 h into differentiation (when the medium was changed), and efficiency of treated cells was expressed as a percentage of untreated cells. Significance was calculated using an unpaired t test (^∗∗∗∗^p < 0.0001, n = 6 independent experiments, mean ± SEM is plotted). (C) WT and *TP53*^−/−^ cells were differentiated either without treatment or with exposure to 5 ppm MMS at 0, 24, and 48 h. Cells were collected at 72 h and monitored for SOX17 expression and cell-cycle phase using DAPI. The SOX17-positive proportion of cells and the proportion of cells in each phase of the cell cycle are shown. Cell-cycle quantification was calculated using the Dean-Jett-Fox model on FlowJo. (D) Alkaline comet analysis was performed on WT and *TP53*^*−/−*^ differentiating cells. Cells were treated with or without 5 ppm MMS at 24 h and collected 2 h later for analysis (n = 2 independent experiments with >400 comets analyzed per condition; p values calculated with Kruskal-Wallis with Dunn's multiple comparisons test, ^∗∗∗∗^p < 0.0001). (E) As for (D) but showing tail moment, ^∗∗∗∗^p < 0.0001.

To explore whether the DNA-damage-induced stabilization of p53 was linked to the failure to acquire markers of DE, we differentiated a *TP53*^−/−^ BOBSC cell line (see Supplemental Experimental Procedures and Figure S3A) with and without exposure to MMS. The untreated *TP53*^−/−^ and wild-type cells differentiated with the same efficiency (Figure 3A). However, *TP53*^−/−^ cells differentiated with a much higher efficiency than the wild-type cell line when treated with MMS during differentiation (Figure 3B), while exhibiting a similar G2/M cell-cycle block (Figure 3C). It is noteworthy that although loss of p53 reduces the fraction of cells in G1 during MMS exposure, consistent with the known role of p53 in the G1/S checkpoint (Smith et al., 2020), the predominant accumulation of cells is in G2/M in both wild-type and, to a greater extent, *TP53*^*−/−*^ cells (Figure 3C). This likely reflects both the chronic, low-dose DNA damage used in these experiments and the significant reliance on S-phase recombination pathways for tolerating the replication-stalling lesions created by MMS (Lundin et al., 2005). Disruption of *TP53* also rescued differentiation when MMS and UV were administered at 24 h only (Figures S3B and S3C). The restoration of efficient differentiation in MMS-treated *TP53*^−/−^ cells was not explained by a change in the number of cell divisions (Figure S3D) and occurred despite similar levels of MMS-induced DNA damage detectable in alkaline comets, compared with wild-type cells (Figures 3D and 3E). Thus, the loss of p53 is sufficient to apparently rescue DE differentiation in the face of DNA damage.

**Figure 4:**
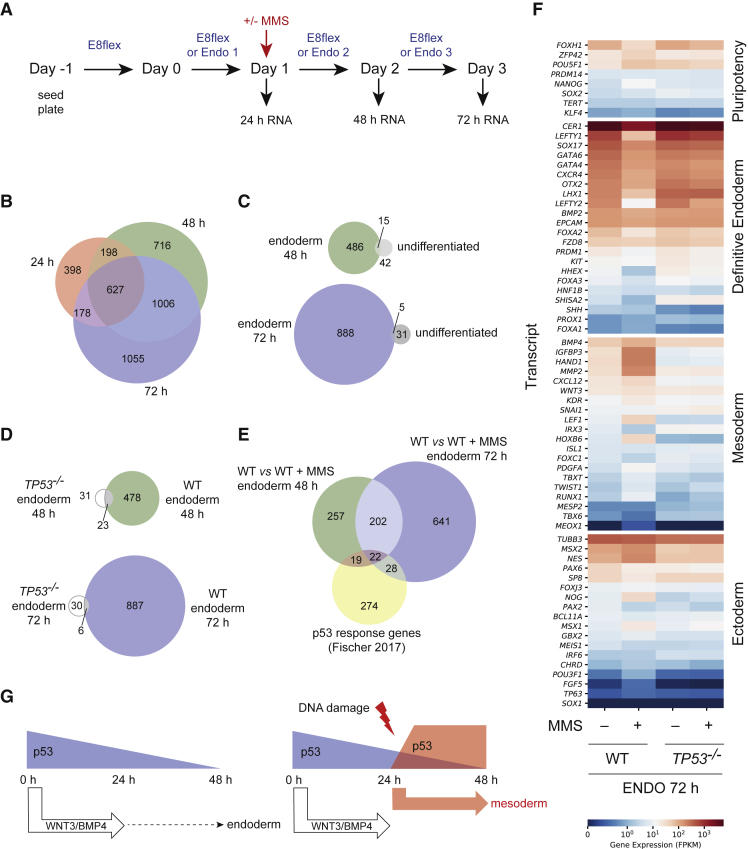
DNA-Damage-Induced Activation of p53 Enforces a Damage-Induced Transcriptional Program that Drives Differentiation toward Mesoderm (A) Schematic to show the workflow of the experimental setup for RNA sequencing. On day −1 WT and *TP53*^*−/−*^ cells were passaged, and on day 0 the experiment was initiated. Undifferentiated cells were cultured, in parallel to the differentiating cells, in Essential 8 Flex (E8flex) medium. RNA was extracted from both cell lines in the undifferentiated state and during differentiation every 24 h (days 1, 2, 3). In parallel, cells were treated with 5 ppm MMS at 24 h and RNA was extracted at days 2 and 3. (B) Gene expression changes during differentiation: differentially expressed genes between untreated WT cells undergoing differentiation and undifferentiated cells collected at 24 h are shown. The overlap between the differences is shown at 24, 48, and 72 h of differentiation. (C) Comparison of the differentially expressed genes after MMS treatment compared with untreated cells in the wild-type cell line: during differentiation (left) and undifferentiated cells (right). The top shows 48 h (24 h after treatment) and the bottom 72 h (48 h after treatment). (D) Comparison of both WT and *TP53*^−/−^ with and without MMS treatment during differentiation. The difference between treated and untreated cells at 48 h (top) and 72 h (bottom) is shown. (E) A Venn diagram to show the overlap of the list of p53-response genes (Fischer, 2017) with those altered during differentiation after MMS treatment. (F) Heatmap to show the expression of different lineage-specific markers and pluripotency markers at 72 h of differentiation in both WT and *TP53*^*−/−*^ cell lines, with and without MMS treatment at 24 h. (G) A model to suggest how DNA damage induces p53 stabilization and this leads to a change in gene expression in these cells, altering the outcome of differentiation. The upregulation of p53 in this context causes upregulation of mesoderm-lineage-specific genes, thus altering the trajectory of differentiation.

To understand the nature of the p53-dependent changes in the DE transcriptional program induced by DNA damage, we performed time-resolved RNA sequencing during endoderm differentiation and in undifferentiated cells, with and without exposure to 5 ppm MMS at 24 h, in both wild-type and *TP53*^*−/−*^ cells (Figure 4A).

### DNA-Damage-Induced p53 Enforces a Transcriptional Program that Drives Differentiation toward Mesoderm

As expected, vast changes in gene expression accompanied endoderm differentiation (Figure 4B and Table S1). Treatment with MMS imposed significant additional changes on gene expression in the cells undergoing endoderm differentiation but had much less effect on undifferentiated cells (Figure 4C). The vast majority of the changes induced by MMS during differentiation were dependent on p53 (Figure 4D). Interestingly, while known p53 targets were transcriptionally upregulated 24 h after exposure to MMS in differentiating cells (Figure S4), only a small percentage of the genes affected are described as robust p53 targets (Figure 4E). This may reflect the strong bias in somatic cell p53 targets in the published literature (Fischer, 2017). Nonetheless, KEGG pathway analysis identifies p53 signaling as the most significantly affected pathway at this time (Table S2). Gene ontology (GO) analysis of the genes differentially expressed at 48 h into differentiation between wild-type treated and untreated cells revealed the most significant terms to be development, morphogenesis, and differentiation (Table S3). This suggests that MMS treatment was specifically affecting differentiation-related pathways. Inclusion of terms involving nervous system development also suggested that MMS treatment may alter lineage specification. At 72 h the significance of the developmental and morphogenesis terms was even more marked (Table S3). The number of genes significantly deregulated by MMS in the *TP53*^*−/−*^ cell line was low (Figure 4D), and thus the GO analysis revealed no significant terms at 48 h, while those that reached significance at 72 h (Table S3) were involved in ion transport, which has previously been linked to p53 function (Mak et al., 2017).

To explore whether DNA damage does indeed redirect differentiation in a p53-dependent manner, we examined changes in expression in key pluripotency and lineage-specific markers (Saili et al., 2019) in each condition (Figure 4F). MMS-treated wild-type cells expressed lower levels of DE markers and higher levels of most mesoderm markers, along with small changes in ectoderm markers. This effect was almost exclusively dependent on p53, demonstrating that the DNA-damage-induced activation of p53 during a critical window of DE specification in hiPSCs alters the trajectory of differentiation.

## Discussion

Our observation that exposure to a low dose of a DNA-damaging agent during a critical window in the differentiation of hiPSCs to DE disturbs the efficiency and fidelity of the differentiation program is consistent with a recent study showing that overproduction of endogenous reactive oxygen species perturbs endoderm differentiation in hiPSCs, reducing *SOX17* expression and creating cells with tumorigenic potential in nude mice (Oka et al., 2020). Our study shows that the DNA-damage-induced redirection of differentiation is entirely dependent on the upregulation of p53. This p53-dependent response occurs without significant purging of damaged cells, suggesting that differentiating hiPSCs lack the “safety net” of an apoptotic response, highlighting the potential risk of damaged and transcriptionally perturbed cells remaining in the population.

Although *Trp53* is dispensable for broadly normal development in the mouse (Donehower et al., 1992), the *Trp53* family, which includes *Trp63* and *Trp73*, has been proposed to act redundantly to initiate mesendoderm differentiation by direct transcriptional regulation (Wang et al., 2017). Interestingly, neither *TP63* nor *TP73* was expressed (Table S4), suggesting that they were unlikely to be substituting for *TP53* in our system. Our observations suggest that the downregulation of p53 during DE differentiation may be necessary to allow the normal transcriptional program to proceed. This is consistent with previous work showing that while p53 is necessary for cells to initiate the EMT, attenuation of its levels by MDM2 is necessary to allow expression of the mesenchymal phenotype (Araki et al., 2010; Chang et al., 2011). Indeed, downregulation of p53 may be directly controlled by this transition, as the EMT factor TWIST1 is able to bind to p53, leading to MDM2-dependent degradation (Piccinin et al., 2012). We suggest that unscheduled stabilization of p53, caused by DNA damage during the EMT, results in a transcriptional perturbation driving differentiation away from DE (Figure 4G). Although the apical transcriptional targets of p53 upregulation in this context remain to be defined, we speculate that the effect could be, at least in part, mediated by extended WNT/BMP signaling (Gertow et al., 2013; Kempf et al., 2016; Lindsley et al., 2006).

It will be important to determine if the effect of p53 stabilization on differentiation after DNA damage that we observe in the lines studied here is generalizable to all human stem cells. However, if p53-dependent reprogramming of endoderm differentiation in response to low doses of DNA damage also occurs during human embryonic development, it may provide a mechanism by which damaged cells could be diverted away from forming germ cells, which are formed from the endodermal lineage. More extensive damage may well lead to increased apoptosis, as has been observed in irradiated mouse embryos (Heyer et al., 2000), and to a disordered embryo that would likely be lost before birth.

## Experimental Procedures

### hiPSC and hESC Culture

Cells were cultured at 37°C, 5% CO_2_ in Essential 8 or Essential 8 Flex medium on 6 well plates coated in Vitronectin-XF (STEMCELL Technologies). Cells were passaged 1:10 every 3–4 days depending on confluency. Cells were detached from the plates by a wash in 1 mL of 0.5 mM EDTA (Thermo Fisher Scientific) followed by incubation in fresh EDTA for 5 min before the EDTA was removed and the cells were blasted with medium to detach them. Cells were maintained as small clumps. Endoderm differentiation was driven as described (Yiangou et al., 2019) (Figure 1B, with full details in Supplemental Experimental Procedures).

### MMS Treatment/UV-C Irradiation

MMS (Sigma) was freshly diluted 1000-fold in cell medium and vortexed before being added to cells at the final concentration. UV-C was delivered in a custom-built shuttered cabinet with a stabilized bulb output measured with a calibrated UV-C meter (UVP, Inc). Prior to irradiation, cell medium was replaced with 1 mL PBS.

### Cell Division and Cell Death Assays

Cell division was monitored with a CellTrace Violet cell proliferation kit (Invitrogen). Cell death was determined using the FITC Annexin V apoptosis detection kit with 7-AAD (Biolegend). Full details of both procedures are provided in the Supplemental Experimental Procedures.

### Flow Cytometry

For intracellular staining, *ca.* 1 × 10^6^ cells were collected, having been detached from the plate with 1 mL Cell Dissociation Buffer (Thermo Fisher Scientific) for 10 min at 37°C. Following centrifugation in a 1.5 mL tube (1,500 × *g*, 4 min), the cell pellet was fixed in 200 μL 1% paraformaldehyde for 10 min at room temperature, repelleted, and resuspended in 200 μL of 90% PBS/10% DMSO and kept at −80°C until preparation for flow cytometry.

Fixed cells were thawed at room temperature for 10 min, pelleted (1,500 × *g*, 4 min), and resuspended in 200 μL 1× BD Perm/Wash buffer. Samples were divided in two to provide an IgG control, pelleted, and resuspended in 100 μL BD buffer. Cells were permeabilized and blocked (15 min, room temperature) and spun down for antibody staining: either primary followed by secondary or conjugated antibody staining. For antibodies used see Supplemental Experimental Procedures.

### Protein Extraction, SDS-PAGE, and Western Blotting

Cells from a single well of a 6 well plate were harvested as described for flow cytometry and protein was extracted using RIPA buffer and standard methods. Full details and antibodies used are given in the Supplemental Experimental Procedures.

### Alkaline Comet Assay

The R&D Systems comet assay kit was used according to the manufacturer's instructions. Further details are in the Supplemental Experimental Procedures. More than 100 comets were analyzed for each replicate of each condition. The CometScore 2.0 software (TriTek Corp.) was used to calculate the average tail moment.

### RNA Extraction and Quantitative Reverse Transcription PCR

RNA extraction was performed using the RNeasy Qiagen RNA extraction kit and reverse transcribed with the Qiagen QuantiTect reverse transcription kit, using 800 ng RNA. qPCR was performed with SYBR Green Mastermix (Applied Biosystems) and a ViiA7 real-time qPCR system (Applied Biosystems). Full details are in the Supplemental Experimental Procedures.

### Library Preparation for RNA Sequencing and RNA-Sequencing Data Analysis

RNA was extracted and libraries were prepared using standard methods (detailed in Supplemental Experimental Procedures). Raw sequencing data were trimmed for adaptor sequences with a minimum quality threshold of 30 using TrimGalore v.0.4.4 (https://github.com/FelixKrueger/TrimGalore). Trimmed reads were aligned to the human genome version GRCh38.87 using TopHat v.2.1.0 (Kim et al., 2013) and quantified per genomic region and differential expression was calculated using Cufflinks v.2.2.1 (Trapnell et al., 2013). Gene lists for Venn diagrams were created using the following cutoffs: fragments per kilobase of transcript per million mapped reads (FPKM) ≥1 for both samples, log_2_ fold change minimum of 1, and significantly different expression taken as calculated from CuffDiff (Trapnell et al., 2013). The Python library Matplotlib (Hunter, 2007) was used to produce Venn diagrams from lists of differentially expressed genes, and Seaborn (https://doi.org/10.5281/zenodo.3629446) was used for generating heatmaps, plotting the mean FPKM per triplicate for each condition.

### Data and Code Availability

The RNA-sequencing data have been deposited in GEO (https://www.ncbi.nlm.nih.gov/geo/) with accession no. GSE146225.

## Author Contributions

C.B.E. performed all experiments and analysis and formed the project. F.J.A. generated the scripts for analysis of RNA-sequencing data and the Venn diagrams and heatmaps; A.C. performed the initial RNA-sequencing alignments and read counts. R.G. and L.V. hosted C.B.E. in the early stages of the project and provided critical guidance on the differentiation system. J.E.S. was responsible for conceptualization and supervision of the project. The paper was written by C.B.E. and J.E.S.

## Supplemental Information

Document S1. Supplemental Experimental Procedures, Figures S1–S4, and Tables S2–S4

Table S1. Differentially Expressed Genes, Related to Figure 4

Document S2. Article plus Supplemental Information
