# Supplementary material for: A p53-Dependent Checkpoint Induced upon DNA Damage Alters Cell Fate during hiPSC Differentiation
Source: Stem Cell Reports. 2020 Sep 3;15(4):827–35. doi: 10.1016/j.stemcr.2020.08.003 (PMC7561492; doi:10.1016/j.stemcr.2020.08.003)
Supplement: Document S1. Supplemental Experimental Procedures, Figures S1–S4, and Tables S2–S4 [file mmc1.pdf]

Stem Cell Reports, Volume *15*

Supplemental Information

A p53-Dependent Checkpoint Induced upon DNA Damage Alters Cell Fate during hiPSC Differentiation

Cara B. Eldridge, Finian J. Allen, Alastair Crisp, Rodrigo A. Grandy, Ludovic Vallier, and Julian E. Sale

Supplemental Information for:

A p53-dependent checkpoint induced upon DNA damage alters cell fate during hiPSC differentiation

Cara B. Eldridge [1], Finian J. Allen [2], Alastair Crisp [1], Rodrigo A. Grandy [3], Ludovic Vallier [3,4,5] and Julian E. Sale [1*]

[1] MRC Laboratory of Molecular Biology, Francis Crick Avenue, Cambridge, CB2 0QH, UK
[2] Department of Chemistry, University of Cambridge, Cambridge, CB2 1EW, UK
[3] Wellcome–MRC Cambridge Stem Cell Institute, Anne McLaren Laboratory, University of Cambridge, Cambridge CB2 0SZ, UK
[4] Department of Surgery, University of Cambridge, Cambridge CB2 0QQ, UK
[5] Wellcome Sanger Institute, Wellcome Genome Campus, Hinxton CB10 1SA, UK

[*] to whom correspondence should be addressed: jes@mrc-lmb.cam.ac.uk

Figure S1 (Related to Figure 1).

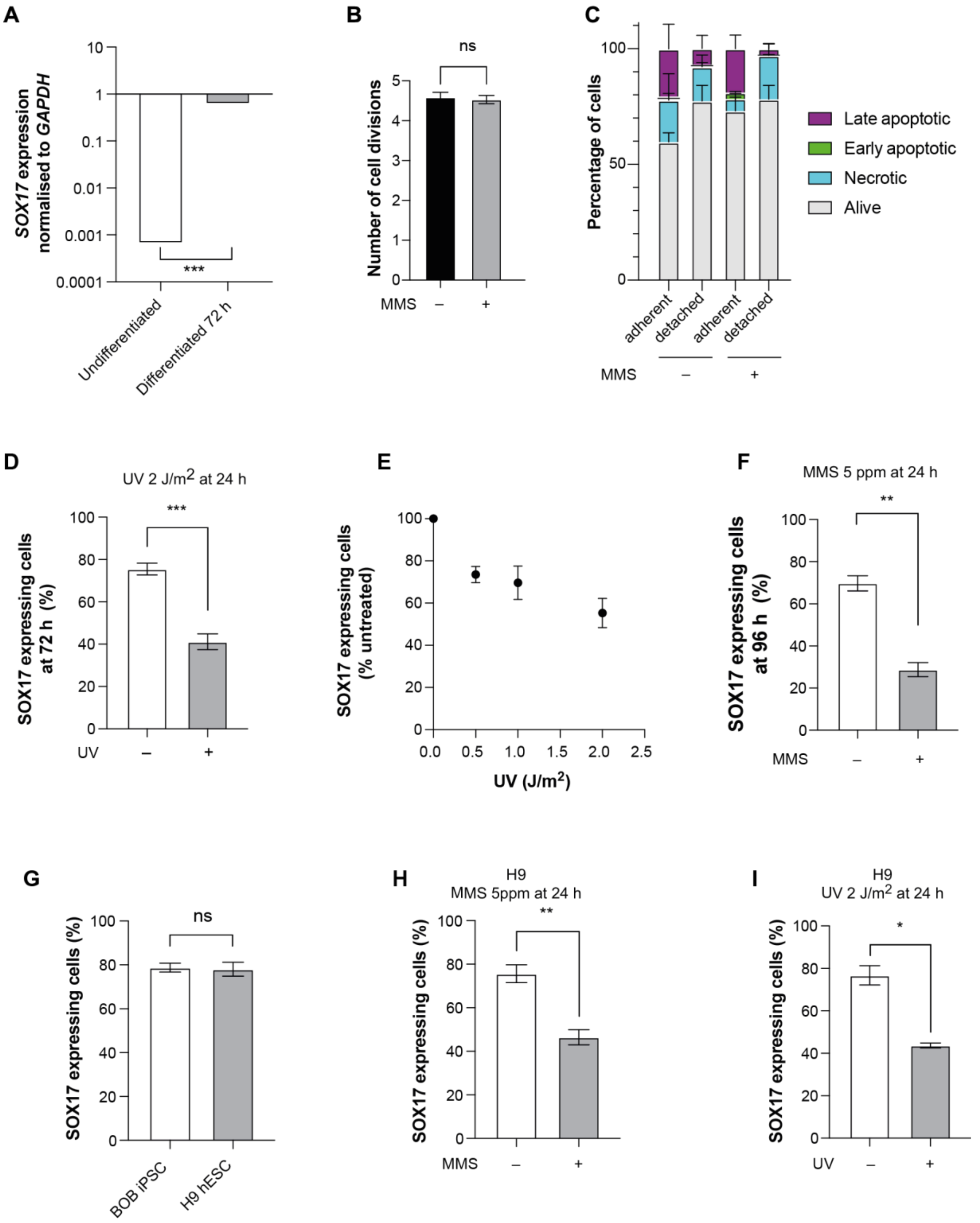

(A) *SOX17* expression calculated using RT-qPCR of undifferentiated and 72 hour differentiating cells normalised to *GAPDH*. (Mean ± SD, n = 2 independent experiments, $p < 0.0004$ using a paired t test.

(B) CellTrace™ Violet was used to analyse the number of cell divisions during differentiation with and without 5 ppm MMS treatment. Cells were incubated with the dye for 20 minutes prior to the set-up of differentiation and then Time 0 cells were collected to analyse the initial fluorescence. Differentiation was then set up in with cells either treated with MMS at 24 hours or untreated, all samples were collected at 72 hours and monitored by flow cytometry. The number of cell cycles was calculated by comparing to the reading at Time 0 (see STAR methods) (n = 4 independent experiments, mean ± SEM, no significant difference was detected between any samples using paired t test).

(C) Viability assay monitoring Annexin V and 7-AAD to measure the proportion of cells undergoing necrosis or apoptosis. Cells were treated with 5 ppm MMS at 24 hours of differentiation and viability was monitored at 48 hours (24 hours later). Adherent cells were those remaining attached to the culture dish whereas detached cells were those that lost adherence. (n = 4 independent experiments, mean ± SD).
(D) Wild-type cells were irradiated with 2 J/m^2 UV-C at 24 hours, or left untreated, and cells were collected at 72 hours to monitor with proportion of SOX17 positive cells (n = 5, p = 0 .0007, paired t-test).
(E) UV-C irradiation dose response in wild-type BOBSC cells. Cells were irradiated at 24 hours and SOX17 expression was monitored at 72 hours (n = 2 independent experiments per time point, mean ± SEM).
(F) Wild-type cells were differentiated with or without 5ppm MMS treatment at 24 hours and differentiation was continued to 96 hours (24 hours longer than usual) to see if cells could catch up with SOX17 expression (n = 4 independent experiments, p = 0.0062 using paired t test).
(G) Wild-type BOBSC hiPS cells were differentiated in parallel with H9 hES cells and the percentage of cells expressing SOX17 at 72 hours was calculated (n = 6 independent experiments, p = 0.8589, unpaired t test mean ± SEM is plotted).
(H) H9 cells were differentiated with or without 5 ppm MMS treatment at 24 hours and the expression of SOX17 at 72 hours was monitored (n = 4 independent experiments, p = 0.0023 using a paired t-test mean ± SEM is plotted).
(I) H9 cells were differentiated with or without 2 J/m^2 UV-C irradiation at 24 hours (n = 3 independent experiments, p = 0.0222 using paired t test mean ± SEM is plotted.)

Figure S2 (Related to Figure 2).

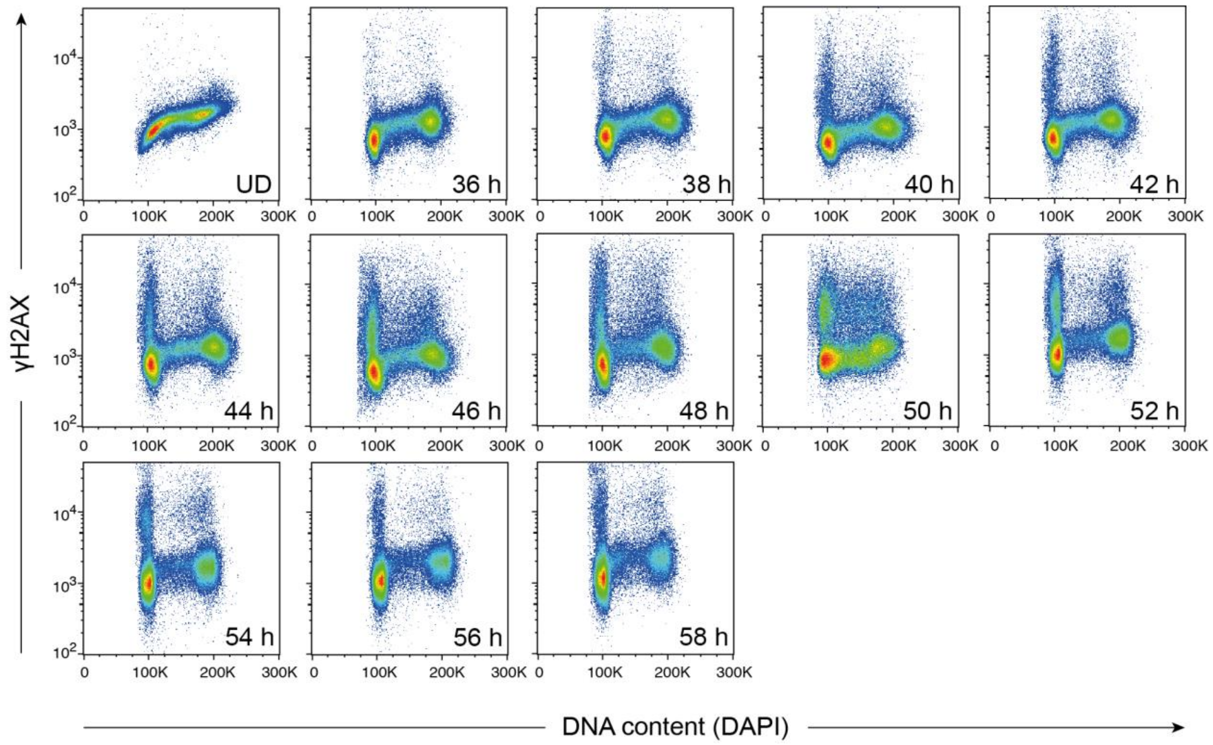

Flow cytometry analysis of the phosphorylation of H2AX during untreated differentiation of wild-type cells. Undifferentiated cells and cells at different times during differentiation were collected and fixed, and then the level of H2AX phosphorylation was monitored with DAPI staining to monitor the position in the cell cycle.

Figure S3 (Related to Figure 3).

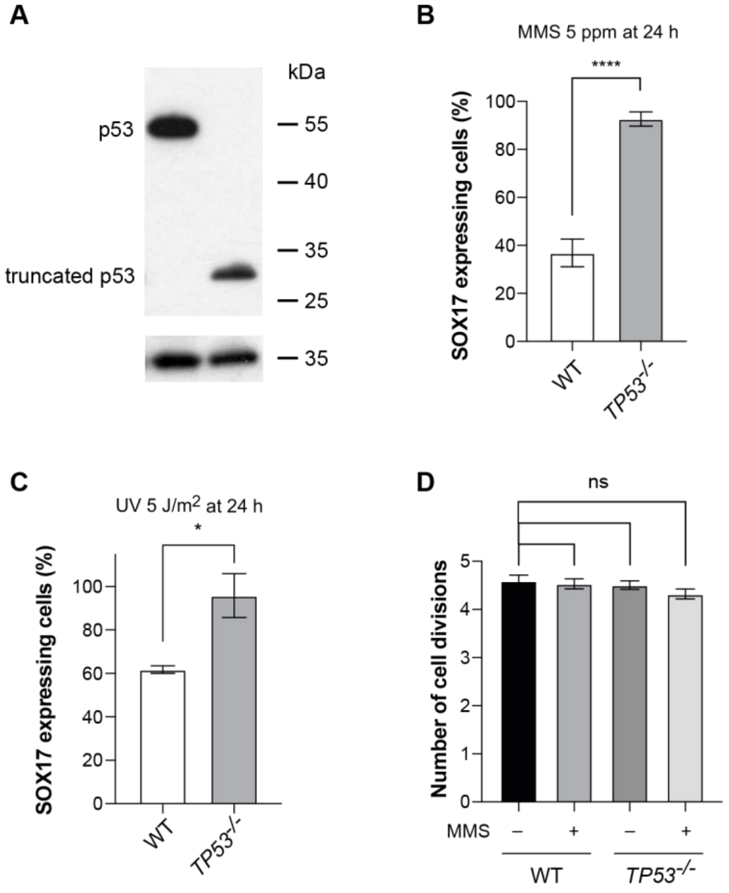

(A) Western blot to show the level of total p53 in the wild-type BOBSC and *TP53*$^{-/-}$ cell lines 5 h after 20 J/m^2 UV-C irradiation to induce p53 stabilisation, β-ACTIN was used as a loading control. The p53 DO-1 antibody binds to amino acids 11-25 of human p53 and therefore detects the N-terminus.
(B) Wild-type and *TP53*$^{-/-}$ cells were differentiated with or without 5 ppm MMS treatment at 24 hours and cells were collected for permeabilised flow cytometry at 72 hours. SOX17 expression after MMS is expressed as a percentage of the untreated level of expression. Wild-type is shown on the left and *TP53*$^{-/-}$ is shown on the right ($n = 6$ independent experiments, $p < 0.0001$ using unpaired t test, mean ± SEM is plotted).
(C) As for B. but using 5 J/m^2 UV-C irradiation at 24 hours ($n = 4$ independent experiments, $p = 0.0161$ using unpaired t test, mean ± SEM is plotted).
(D) S1C but with *TP53*$^{-/-}$ cells added ($n = 4$, independent experiments; no significance was detected between any samples using one-way ANOVA).

Figure S4 (Related to Figure 4).

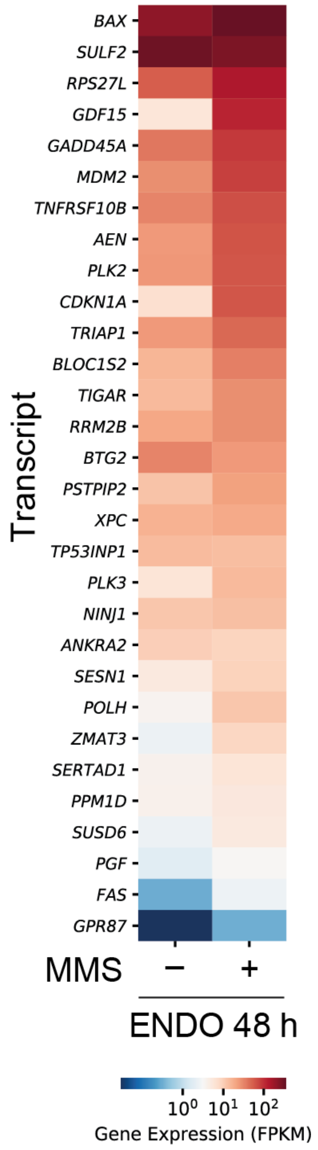

A heatmap to show the expression levels of p53-response genes, expressed as mean FPKM values, in the wild-type cell line at 48 hours, with and without MMS treatment at 24 hours.

Supplemental Tables

Supplemental Table 1 (Related to Figure 4).
Differentially expressed genes.
See separate Excel spreadsheet.

Supplemental Table 2 (Related to Figure 4).

Term ID	Description	Adjusted p-value	Count in selected genes	Count in all genes
KEGG:04115	p53 signaling pathway	0.000694	11	72
KEGG:04371	Apelin signaling pathway	0.0358	12	137
KEGG:05200	Pathways in cancer	0.0542	27	526
KEGG:05205	Proteoglycans in cancer	0.0542	14	199
KEGG:04151	PI3K-Akt signaling pathway	0.0595	20	353
KEGG:05216	Thyroid cancer	0.104	5	37
KEGG:05218	Melanoma	0.104	7	72
KEGG:04510	Focal adhesion	0.19	11	199
KEGG:04060	Cytokine-cytokine receptor interaction	0.19	15	292
KEGG:05225	Hepatocellular carcinoma	0.19	10	167

KEGG pathway analysis using genes differentially expressed in wild-type untreated compared to MMS treated cells Kyoto Encyclopedia of Genes and Genomes **(**KEGG) analysis of wild-type 48 h differentiating cells treated with and without MMS. Benjamini-Hochberg multiple testing correction was used to calculate probabilities. Only the top two pathways were significant.

Supplemental Table 3 (Related to Figure 4).

Wild type				
48 hours				
Term ID	Description	Adjusted p-value	Count in selected genes	Count in all genes
GO:0048856	anatomical structure development	6.30×10^{-19}	218	5793
GO:0032502	developmental process	8.68×10^{-19}	227	6212
GO:0007275	multicellular organism development	1.04×10^{-16}	200	5321
GO:0048731	system development	1.17×10^{-15}	183	4760
GO:0009653	anatomical structure morphogenesis	1.87×10^{-15}	123	2598
GO:0032501	multicellular organismal process	2.05×10^{-15}	245	7414
GO:0007399	nervous system development	3.60×10^{-15}	113	2299
GO:0030154	cell differentiation	6.24×10^{-15}	163	4088
GO:0023051	regulation of signaling	9.58×10^{-15}	146	3482
GO:0010646	regulation of cell communication	9.58×10^{-15}	145	3447
72 hours				
GO:0009653	anatomical structure morphogenesis	2.31×10^{-32}	236	2598
GO:0048856	anatomical structure development	5.53×10^{-32}	395	5793

GO:0007275	multicellular organism development	$5.53x10^{-32}$	373	5321
GO:0032502	developmental process	$1.56x10^{-29}$	407	6212
GO:0009887	animal organ morphogenesis	$1.79x10^{-29}$	126	969
GO:0048731	system development	$1.42x10^{-26}$	332	4760
GO:0048513	animal organ development	$4.95x10^{-25}$	262	3428
GO:0032501	multicellular organismal process	$9.50x10^{-25}$	445	7414
GO:0048646	anatomical structure formation involved in morphogenesis	$8.86x10^{-23}$	126	1144
GO:0009888	tissue development	$3.20x10^{-22}$	173	1926
TP53$^{-/-}$				
72 hours				
GO:0008324	cation transmembrane transporter activity	0.0119	7	664
GO:0046873	metal ion transmembrane transporter activity	0.0119	6	457
GO:0022803	passive transmembrane transporter activity	0.0119	6	470
GO:0015267	channel activity	0.0119	6	469
GO:0005261	cation channel activity	0.0119	5	320
GO:0015075	ion transmembrane transporter activity	0.0292	7	897
GO:0022890	inorganic cation transmembrane transporter activity	0.0292	6	605
GO:0015077	monovalent inorganic cation transmembrane transporter activity	0.0292	5	392
GO:0005215	transporter activity	0.0301	8	1251
GO:0022842	narrow pore channel activity	0.0312	2	19

GO analysis of differentially expressed genes in wild-type and *TP53*$^{-/-}$ cells with and without MMS treatment, at 48 and 72 hours of differentiation Gene ontology (GO) analysis of the untreated cell line compared to the MMS treated cell line at 48 hours and 72 hours. PANTHER was used to perform enrichment analysis and Benjamini-Hochberg multiple testing correction was used to calculate probabilities. No significant terms were seen for *TP53*$^{-/-}$ at 48 hours using $p < 0.05$.

Supplemental Table 4 (Related to Figure 4).

	TP53	***TP63***	***TP73***
Undifferentiated	107.7761	0.069823	0.765466
24 h differentiated	85.87637	0.119782	0.594662
48 h differentiated	84.11267	0.065392	0.324827
72 h differentiated	101.423	0.118819	0.70977

Expression levels of the *TP53* family during differentiation. Gene expression of members of the *TP53* family expressed as average FPKM (n = 3) in wild-type untreated cells during differentiation. The undifferentiated sample is the 24 h undifferentiated sample. An FPKM > 1 was used as the threshold for detection of expression.

Supplemental Experimental Procedures

hiPS and hES cells

BOBSC cells (Andersson-Rolf et al., 2017), a derivative of cA1ATD cells (Yusa et al., 2011), were obtained from the Sanger Centre. p53-deficient BOBSC lines were generated by the COMSIG (Causes of Mutational SIGnatures) project, funded by the Wellcome Trust, using a CRISPR/Cas9 strategy in which one allele was disrupted by gene targeting and the other by error-prone repair.

**Cell line**	**Sanger HTGT plate code**	**Barcode**	**Targeting**
WT cA1ATD (BOBSC)		BOBSC-T6/8_B1	n/a
TP53 KO	HUFP0007_3_A_H04	1095765195	Exon 6

The WA09 (H9) hES cells were obtained from WiCell via Dr. Madeline Lancaster, MRC LMB, Cambridge. They were cultured as for the BOBSC cells.

Endoderm Differentiation protocol

Cells were passaged 1:8 onto Vitronectin-XF (StemCell Technologies) coated six well plates one day prior to setting up endoderm differentiation. On day one of differentiation, cell medium was changed to CDM-PVA supplemented with 100 ng/mL Activin A (R&D), 80 ng/mL FGF2 (R&D), 10 ng/mL BMP4 (R&D), 10 µM PI3K inhibitor LY294002 (Promega) and 3 µM GSK3i CHIR99021 (Tocris). On day two, cell medium was replaced with CDM-PVA supplemented with Activin A, FGF2, BMP4 and LY294002. CDM-PVA consisted of 50% Ham's F-12 (Gibco) and 50% IMDM (Gibco) supplemented with 1 g/L PVA (Sigma), 1 mM concentrated Lipids (Life technologies), 0.5 mM Thioglycerol (Sigma), 15 µg/mL Transferrin (Roche) and 7 µg/mL Insulin (Roche). The PVA solution was first made up in ultrapure water by heating to 90˚C with stirring, continuing overnight while cooling to ensure the powder was dissolved. On day three, the medium was replaced with RPMI+ supplemented with 100 ng/mL Activin A and 80 ng/mL FGF2. RPMI was supplemented with 1 mM NEM-NEEA (Gibco) and B27 supplement (Yiangou et al., 2019).

Cell division assay

Cell divisions were monitored with CellTrace™ Violet Cell Proliferation Kit (Invitrogen™). Cells were loaded with 5 µM CellTrace™ reagent in PBS for 20 min at 37˚C. The cells were washed three times with warm medium and incubated until analysis. Cells should lose half of the fluorescence at each cell division. Therefore, comparing the fluorescence of the cells collected at the start and end of differentiation, the number of cell divisions can be quantified using the equation below.

$$N = \frac{\log\left(\frac{Co}{Ct}\right)}{\log 2}$$

Cell death assay

Cell death was determined using the FITC Annexin V Apoptosis Detection Kit with 7-AAD (Biolegend®). Both adherent and non-adherent cell fractions were collected and pelleted at 300 *g* for 4 min. Cells were washed twice in Cell Staining Buffer and then resuspended in 100 µL Annexin V Binding Buffer per well of a six well plate. The cells were transferred to a flow cytometry tube and 5 µL FITC Annexin V and 5 µL 7-AAD was added. The cells were vortexed and incubated in the dark at room temperature for 15 min). 400 µL Annexin V Binding Buffer was added to each tube and the cells were analysed using flow cytometry.

Antibody staining of permeabilised cells

For monitoring γH2AX, permeabilised cells were resuspended in 100 µL anti-γH2AX antibody (1/500) (Merck Millipore 05-636) (4°C, overnight) or IgG control without antibody. Cells were spun down (1500 *g*, 4 min), washed in BD buffer twice and resuspended in secondary antibody (1/200) (Invitrogen Alexa Fluor®) (1 h, RT, dark). Cells were spun down and washed, as above, and resuspended in 400 µL PBS/BSA 0.5% with 1 µg DAPI per sample and protected from light until analysis. For staining SOX17 and EOMES, directly conjugated antibodies were used, as below. Cells were resuspended in 100 µL BD buffer containing 4 µL conjugated antibody, or appropriate isotype control, per reaction and incubated in the dark (30 min, RT). Cells were washed

once in BD buffer, spun down (1500 *g*, 4 min) and resuspended in 400 µL PBS/BSA 0.5% with 1 µg DAPI per sample for analysis.

Protein	Conjugated Primary Antibody
SOX17	BD Pharmingen™ 562205 Alexa Fluor® 488 Ms
EOMES	Invitrogen 50-4877-42 eBioscience™ eFluor® 660 Ms

All flow cytometry was performed on a BD LSRFortessa™, which is equipped with 405 nm, 488 nm, 561 nm and 640 nm lasers with analysis using FlowJo®, LLC, initially gating out debris using FSC/SSC and then gating for single cells using DAPI staining V-450A/V-450H.

Protein extraction, SDS-PAGE and western blotting

Cells from a single well of a six well plate were harvested as described for flow cytometry. Following two washes in 500 µL PBS, cells were resuspended in 100 µL per 500,000 cells RIPA buffer (Cell Signalling) containing Benzonase® nuclease (1/1000) and 1X Halt™ Protease and Phosphatase inhibitor cocktail (Thermo Scientific). Eppendorfs containing cells and buffer were rotated slowly (4˚C, 1 h) and then transferred to a cold centrifuge (16,000 *g*, 4˚C, 30 min). The supernatant (protein) was transferred to a clean tube, being careful not to disturb the DNA pellet at the bottom. 5X SDS-PAGE sample buffer (Sigma) was added to 1X and the protein was boiled (95˚C, 5 min). Protein was stored at -20˚C until use. Protein was thawed on ice and then was run on a NuPAGE® Bis-Tris 4-12% precast gel (Thermo Fisher) in 1X MOPS buffer (50 mM MOPS, 50 mM Tris, 0.1% SDS, 1 mM EDTA, pH 7.7) at 120 V for 2 h. Semi-dry transfers to nitrocellulose were performed using the iBlot® 2 system (Thermo Fisher) using iBlot® 2 transfer stacks at 25 V for 7 min. The membrane was put into deionised water, cut to size and stained with Ponceau S (15 s, RT, rotating) to check sample loading and transfer efficiency. The membrane was washed twice and blocked in 5% milk (Marvel) in 1X TBST (150 mM NaCl, Tris HCl pH7.4 10 mM, 0.1% tween) (1 h, RT, rotating).

Primary antibodies were added as stated in the table below. Membranes were incubated with primary antibody overnight, rotating at 4˚C. The membrane was washed three times for 5-15 minutes while rotating at room temperature with 1X TBST. The membrane was then blotted with secondary antibody (1 h, RT, rotating). All secondary antibodies were HRP-conjugated (Dako P0447-9) used at 1/5000 dilution. The secondary antibody was washed as for the primary antibody and signal revealed with Millipore Luminata Crescendo reagent.

Antibodies

Protein epitope	Dilution	Catalogue no.	Manufacturer	Species
β-ACTIN	1:20,000	Ab8227	Abcam	Rabbit
pCHK1 (Ser-345) (133D3)	1:1000	113D3	CST	Rabbit
CHK1	1:1000	Ab40866	Abcam	Rabbit
pCHK2 (Thr-68)	1:1000	2661	CST	Rabbit
CHK2	1:50,000	Ab109413	Abcam	Rabbit
γH2AX (Ser-139) JBW301	1:5000	05-636	Merck Millipore	Mouse
H2AX total	1:500	SC-517336	Santa Cruz	Mouse
p-p53 (Ser15)	1:1000	9284	CST	Rabbit
p53 (DO-1)	1:1000	Ab1101	Abcam	Mouse
PC10 (PCNA)	1:10,000	SC-56	Santa Cruz	Mouse
MDM2	1:1000	Ab259265	Abcam	Rabbit
pRPA32 (Ser-33)	1:1000	A300-246A	Bethyl	Rabbit
RPA32	1:1000	Ab2175	Abcam	Mouse

Alkaline comet assay

The R&D systems comet assay kit was used according to the manufacturer's instructions. Briefly, cells were washed in ice cold DPBS and resuspended at $1x10^5$ cells/ mL in ice cold DPBS. 50 µL cells were added to 500 µL warm low melting point agarose and mixed before pipetting onto two wells of the comet slide. Slides were left at 4˚C in the dark for one hour and then kept in cold lysis buffer at 4°C for six days. Slides were immersed in alkaline unwinding solution (200 mM NaOH, 1 mM EDTA) (20 min, 4˚C) prior to electrophoresis. Cells were electrophoresed in the same solution (32 V, 300 mA, 30 min). Slides were washed twice in ultrapure water followed by 70% ethanol for 20 minutes. Slides were dried and stained with SybrGold before analysis on the microscope.

RNA extraction and quantitative reverse transcription PCR (RT-qPCR)
Medium was aspirated from adherent cells and replaced with 600 µL RLT buffer containing β-mercaptoethanol. Cells were transferred to sterile Eppendorf tubes and stored at -80˚C or processed further immediately. Extraction was performed according to the manufacturer's instructions, but without the initial spin to remove debris. RNA was eluted into 30 µL nuclease free water and 1 µL was analysed on the nanodrop to check RNA concentration and for contamination with DNA or protein. RNA was stored at -80˚C. RNA was reverse transcribed to DNA using Qiagen QuantiTect® reverse transcription kit using 800 ng RNA. The initial gDNA 'wipeout' step was performed (42˚C, 2 min) and the reverse transcription (42˚C, 25 min). cDNA was stored at -20˚C for up to 48 hours before qPCR was performed.

The qPCR master mix was made up of 50% SYBR® Green Mastermix (Applied Biosystems), 10 µM Fwd and 10 µM Rv primer and 10% diluted cDNA in DEPC. Reactions were set up on a 96 or 384-well optical plate in 20 µL volumes in triplicate and sealed with optical film. The plate was spun down (3220 *g*, 1 min) and run on a ViiA7™ real-time qPCR system (Applied Biosystems) for 45 cycles (hold stage: 50˚C, 2 min and 95˚C, 10 min and PCR stage: 95˚C, 15 s and 60˚C, 1 min).

**Transcript**	**Fwd Primer: 5'-3'**	**Rv Primer: 5'-3'**
GAPDH	TCACCAGGGCTGCTTTTAACT	GACGGTGCCATGGAATTTGC
SOX17	CGCACGGAATTTGAACAG TA	GGATCAGGGACCTGTCACAC

Library preparation for RNA sequencing
RNA was extracted and quantified as above before running on an Agilent RNA Pico 6000 chip using an Agilent 2100 Bioanalyzer. RNA with a RIN score above 7 was used to generate RNA libraries. 750 ng of RNA was diluted into 50 µL DEPC water and kept on ice. RNA libraries were prepared using the NEBNext UltraII RNA library preparation kit (New England Biolabs E7770) and PolyA tail enrichment with eight PCR cycles and the NEBNext Oligos 1-24 and then stored at -20˚C until analysis. One µg of each constructed library was run on an Agilent 2100 Bioanalyzer High Sensitivity DNA chip and the electrogram was analysed for shape of the graph and the average size of each library (bp): the library was checked for primer (~80 bp) and adapter (~128 bp) contamination and any libraries with high primer/adapter content were re-purified. The libraries were next quantified using the KAPA Library Quantification Kit for Illumina® (Roche). Libraries were serially diluted to 1/100000 and 1/1000000 in 10 mM Tris-HCl pH 8.0 and quantified in triplicate as stated in the protocol using the Rox-low buffer for the ViiA7 qPCR system (Thermo Fisher Scientific Melt-curves for the libraries were also analysed to assess the quality of the libraries. Average C_Ts were transferred to the KAPA quantification excel spreadsheet with standard values and the concentration of each library was calculated. After quantification, libraries created with different NEBNext Oligos were pooled to a total concentration of 20 nM in 10 mM Tris-HCl pH 8.0. The pooled library was run on a High Sensitivity DNA chip, as above, and the average size of the library calculated before sending to sequencing. 20 µL of pooled library was sequenced on a HiSeq4000 machine with single end reads.
