# Supplementary material for: A p53-Dependent Checkpoint Induced upon DNA Damage Alters Cell Fate during hiPSC Differentiation
Source: Stem Cell Reports. 2020 Sep 3;15(4):827–35. doi: 10.1016/j.stemcr.2020.08.003 (PMC7561492; doi:10.1016/j.stemcr.2020.08.003)
Supplement: Document S2. Article plus Supplemental Information [file mmc3.pdf]

Stem Cell Reports
Report

OPEN ACCESS

A p53-Dependent Checkpoint Induced upon DNA Damage Alters Cell Fate during hiPSC Differentiation

Cara B. Eldridge,[1] Finian J. Allen,[2] Alastair Crisp,[1] Rodrigo A. Grandy,[3] Ludovic Vallier,[3,4,5] and Julian E. Sale[1,*]
[1]MRC Laboratory of Molecular Biology, Francis Crick Avenue, Cambridge CB2 0QH, UK
[2]Department of Chemistry, University of Cambridge, Cambridge CB2 1EW, UK
[3]Wellcome–MRC Cambridge Stem Cell Institute, Anne McLaren Laboratory, University of Cambridge, Cambridge CB2 0SZ, UK
[4]Department of Surgery, University of Cambridge, Cambridge CB2 0QQ, UK
[5]Wellcome Sanger Institute, Wellcome Genome Campus, Hinxton CB10 1SA, UK
*Correspondence: jes@mrc-lmb.cam.ac.uk
https://doi.org/10.1016/j.stemcr.2020.08.003

SUMMARY

The ability of human induced pluripotent stem cells (hiPSCs) to differentiate *in vitro* to each of the three germ layer lineages has made them an important model of early human development and a tool for tissue engineering. However, the factors that disturb the intricate transcriptional choreography of differentiation remain incompletely understood. Here, we uncover a critical time window during which DNA damage significantly reduces the efficiency and fidelity with which hiPSCs differentiate to definitive endoderm. DNA damage prevents the normal reduction of p53 levels as cells pass through the epithelial-to-mesenchymal transition, diverting the transcriptional program toward mesoderm without induction of an apoptotic response. In contrast, *TP53*-deficient cells differentiate to endoderm with high efficiency after DNA damage, suggesting that p53 enforces a "differentiation checkpoint" in early endoderm differentiation that alters cell fate in response to DNA damage.

INTRODUCTION

In vitro differentiation of human induced pluripotent stem cells (hiPSCs) and embryonic stem cells (hESCs) to definitive endoderm (DE) is induced by activation of the Activin/Nodal, FGF, BMP4, and WNT signaling pathways in concert with inhibition of phosphoinositide 3-kinases (Vallier et al., 2009). Downregulation of the core pluripotency transcription factors *SOX2*, *NANOG*, and *POU5F1* increases expression of *EOMES* (Teo et al., 2011), which drives a transition through a primitive streak-like stage (Arnold et al., 2008). The cells then undergo the epithelial-to-mesenchymal transition (EMT) and acquire markers of DE, including *SOX17* (Kanai-Azuma et al., 2002) and *FOXA2* (Dufort et al., 1998).

p53 is central to the cellular response to DNA damage (Williams and Schumacher, 2016). In somatic cells, p53 is maintained at a low level through degradation induced by the E3 ubiquitin ligase MDM2 (Michael and Oren, 2003). In response to DNA damage, a well-described signaling cascade leads to ATM-dependent phosphorylation of p53 and its stabilization (Banin et al., 1998; Canman et al., 1998). Activated p53 then drives a transcriptional program leading to cell-cycle arrest to facilitate DNA repair and, if unsuccessful, senescence or apoptosis (Shiloh and Ziv, 2013).

In mouse embryonic stem cells p53 activation can lead to differentiation through suppression of the pluripotency factor *Nanog* (Lin et al., 2005) and modulation of WNT signaling (Lee et al., 2010). This leads to damaged cells being removed from the stem cell pool, limiting their capacity to propagate genetic defects (Lin et al., 2005). Nonetheless, *Trp53*-null mice largely develop normally (Donehower et al., 1992), although female mice are highly susceptible to exencephaly (Armstrong et al., 1995; Sah et al., 1995). In contrast, in *Xenopus* embryos depleted of p53, mesoderm differentiation is inhibited (Cordenonsi et al., 2003) and gastrulation is not completed (Wallingford et al., 1997). These differences may be explained at least in part by the availability of other p53 family members in mammalian cells, p63 and p73, which together with p53 are important for coordination of signaling pathways controlling mesendoderm differentiation (Wang et al., 2017) by inducing expression of genes, including members of the *Wnt* and *Fzd* families (Lee et al., 2010). WNT activates TCF3, which, with SMAD2/3, binds to enhancers of mesendoderm genes and activates their transcription (Wang et al., 2017).

Elevated p53 levels in the early embryo decline significantly during embryonic development (Schmid et al., 1991). Mice lacking one of the p53 regulators *Mdm2* or *Mdm4* do not survive embryonic development, and this can be rescued by concurrent disruption of *Trp53* (Jones et al., 1995; Montes de Oca Luna et al., 1995; Parant et al., 2001). Conversely, overexpression of *Trp53* perturbs renal differentiation, further suggesting that tight control of p53 is required for successful development (Godley et al., 1996). Despite this work on the roles of p53 in both differentiation and the response of stem cells to DNA damage, surprisingly little is known about the p53-dependent response to DNA damage in a differentiating stem cell. Here we address this question using an iPSC model of human endoderm differentiation.

© 2020 MRC Laboratory of Molecular Biology.
This is an open access article under the CC BY license (http://creativecommons.org/licenses/by/4.0/).

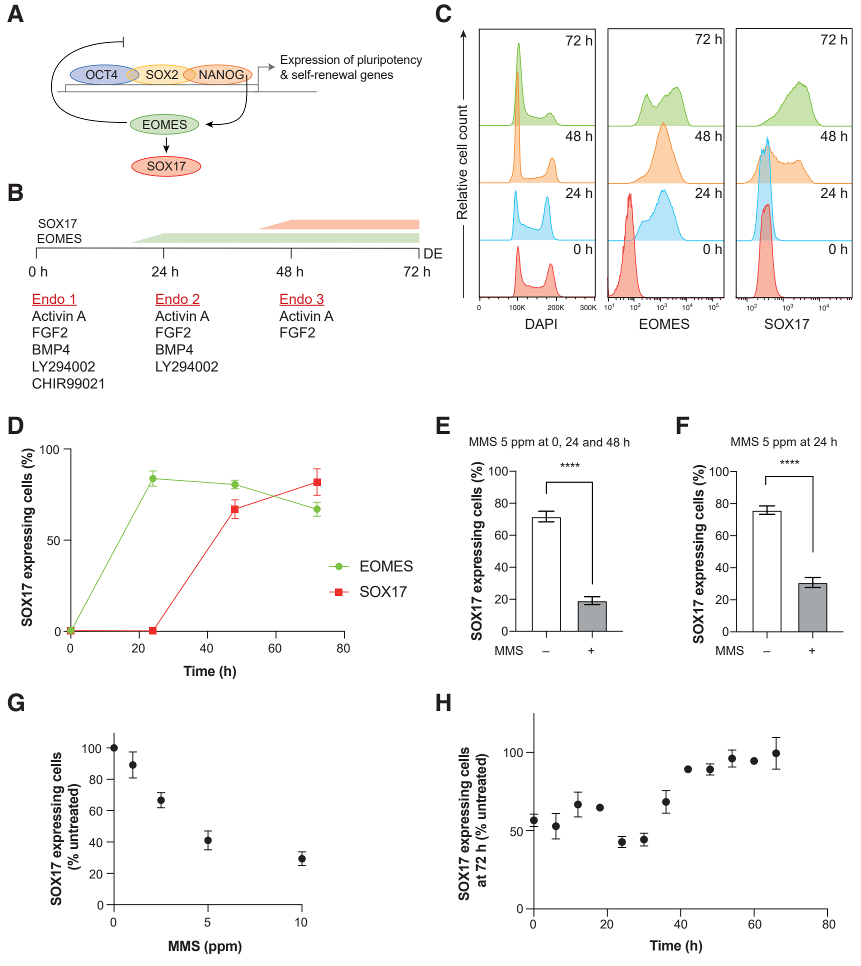

Figure 1. DNA Damage Inhibits Endoderm Differentiation in hiPSCs

(A) Highly simplified genetic circuit controlling differentiation to DE.

(B) Scheme for *in vitro* endoderm differentiation over 72 h.

(C) Permeabilized flow cytometry to monitor expression of EOMES (center) and SOX17 (right) during differentiation to DE. DNA content (DAPI, left) shows the change in the cell-cycle profile during differentiation, with a marked shift toward G1.

(D) Kinetics of EOMES and SOX17 expression during DE differentiation (n = 4 indpendent experiments, mean ± SEM is plotted).

(E) Inhibition of DE differentiation by exposure to MMS throughout differentiation. MMS (5 ppm) was administered at 0, 24, and 48 h (n = 8 independent experiments, ****$p < 0.0001$ using a paired t test, mean ± SEM is plotted).

(F) Inhibition of DE differentiation by exposure to a single dose of 5 ppm MMS at 24 h (n = 12 independent experiments, ****$p < 0.0001$ using a paired t test, mean ± SEM is plotted).

(G) Dose-dependent inhibition of DE differentiation by MMS treatment at 24 h (n = 5 independent experiments, mean ± SEM is plotted).

(H) Cells were treated with 5 ppm MMS every 6 h during differentiation and the efficiency of differentiation at 72 h was monitored (n ≥ 2 independent experiments for each time point, mean ± SEM is plotted).

RESULTS

DNA Damage Decreases the Efficiency of hiPSC Differentiation to DE

We employed the BOBSC hiPSC line (Andersson-Rolf et al., 2017), a derivative of cA1ATD (Yusa et al., 2011), and an established protocol for driving DE differentiation over 72 h (Yiangou et al., 2019) (Figures 1A and 1B). Differentiation was initially monitored using flow cytometry on permeabilized cells with antibodies against the mesendoderm marker EOMES and against SOX17, which marks DE (Arnold et al., 2008; Kanai-Azuma et al., 2002). The proportion of cells expressing SOX17 at 72 h thus provides a simple assay to monitor the efficiency of differentiation (Figures 1C and 1D). Detection of SOX17 by flow cytometry closely mirrored changes in mRNA levels during differentiation (Figure S1A).

To assess the impact of DNA damage on endoderm differentiation, we treated cells with 5 ppm methyl methanesulfonate (MMS) at 0, 24, and 48 h after initiating differentiation (Figure 1B). MMS generates 7-methylguanine and 3-methyladenine in DNA (Beranek, 1990), which can lead to replication stalling and DNA damage (Lundin et al., 2005). It has a half-life of about 4.5 h in aqueous solution (PubChem: CID 4156 https://pubchem.ncbi.nlm.nih.gov/compound/4156). MMS treatment resulted in a significant reduction in SOX17-expressing cells at 72 h (Figure 1E). A single dose of MMS at 24 h (Figure 1B) had a similar effect (Figure 1F) and did not alter the number of cell divisions (Figure S1B) or cell viability (Figure S1C). Likewise, a single low dose of UV-C (2 J/m^2) at 24 h reduced the proportion of SOX17-expressing cells at 72 h (Figure S1D). Furthermore, the decrease in efficiency of differentiation was proportional to the dose of MMS (Figure 1G) and UV (Figure S1E). Importantly, the reduction in SOX17-positive cells at 72 h following damage was not simply the result of differentiation delay, as continuing to 96 h did not increase the proportion of cells expressing SOX17 (Figure S1F).

In order to ensure that the damage-induced decrease in the efficiency of differentiation was not a specific feature

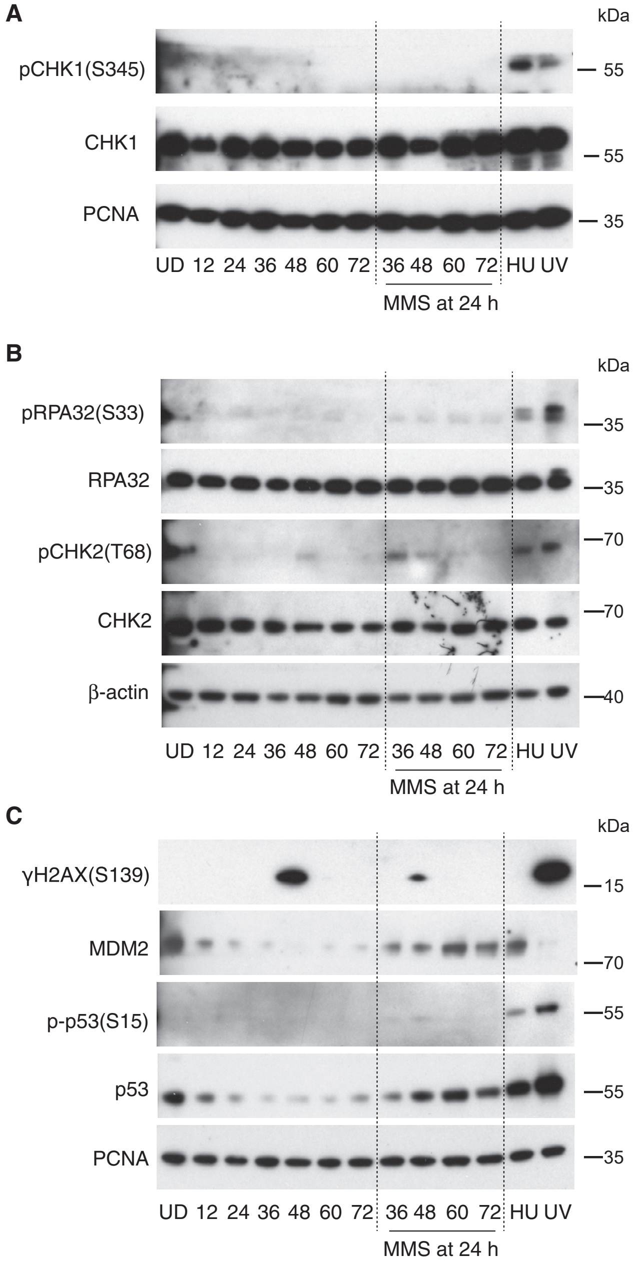

Figure 2. DNA Damage during Early Endoderm Differentiation Prevents Reduction of p53 Levels

Western blot analyses of changes to the DNA-damage response during differentiation, with and without 5 ppm MMS treatment at 24 h. Protein was collected from whole-cell lysates every 12 h. Positive control samples were collected for protein 5 h after treatment, including 2 mM HU and 20 J/m^2 UV-C.

(A) Phospho-CHK1 (Ser-345) and total CHK1, with PCNA as a loading control.

(B) Phosphorylation of RPA32 (Ser-33), total RPA, phosphorylation of CHK2 (Thr-68), total CHK2, and β-ACTIN as a loading control.

(C) Phospho-H2AX (Ser-139), total MDM2, phospho-p53 (Ser-15), and total p53. PCNA is used as a loading control.

of the BOBSC hiPSCs, we also assessed differentiation of the H9 hESC line (Thomson et al., 1998), which had a differentiation efficiency similar to that of BOBSC (Figure S1G). Treatment with both MMS and UV irradiation at 24 h likewise significantly decreased the number of cells expressing SOX17 at 72 h (Figures S1H and S1I), confirming that the effect of DNA damage on differentiation is likely to be generalizable.

We next determined the effect of DNA damage on differentiation efficiency as a function of time of exposure during the protocol. A single dose of 5 ppm MMS was administered at different times during the differentiation protocol and the proportion of cells expressing SOX17 at 72 h measured (Figure 1H). MMS had a maximal inhibitory effect on differentiation when administered in a window between ~20 and 34 h. Treatment after 40 h had very little effect on the outcome of differentiation, when a proportion of cells had begun expressing SOX17. This suggested that once the cells have committed to DE, the outcome cannot be altered by DNA damage.

A Fall in p53 Level during Early Endoderm Differentiation Is Prevented by DNA Damage

To understand the role of DNA-damage-response (DDR) pathways throughout differentiation, we monitored key features of the DDR by western blotting (Figure 2). The total levels of key DDR kinases CHK1 and CHK2, as well as one of their substrates, RPA, did not change during the differentiation protocol (Figures 2A and 2B). However, p53 levels fell as differentiation proceeded (Figure 2C), but were increased following treatment with 5 ppm MMS at 24 h (Figure 2C). Interestingly, while the low dose of MMS used in these experiments stabilized p53 and induced MDM2 expression, it was accompanied by minimal or no phosphorylation of the DNA-damage markers CHK1, CHK2, and RPA; H2AX; or p53 itself. A somewhat surprising observation in this context was a spike of phosphorylation of the histone variant H2AX around 50 h into differentiation (Figure S2). Phosphorylated H2AX (γH2AX) is frequently used as a surrogate marker for DNA double-strand breaks (Rogakou et al., 1998) and was therefore unexpected during normal differentiation. The observed spike of γH2AX was not induced by exogenous DNA damage; indeed, it was attenuated by it (Figure 2C) and was also not accompanied by significant DNA-damage signaling (Figure 2). We speculate that it may be related to the EMT through which the cells pass at this time (Teo et al., 2011). Indeed, H2AX phosphorylation during the EMT in cancer cell line models has previously been shown to play a role in transcriptional regulation of the vast gene expression changes that occur (Singh et al., 2015).

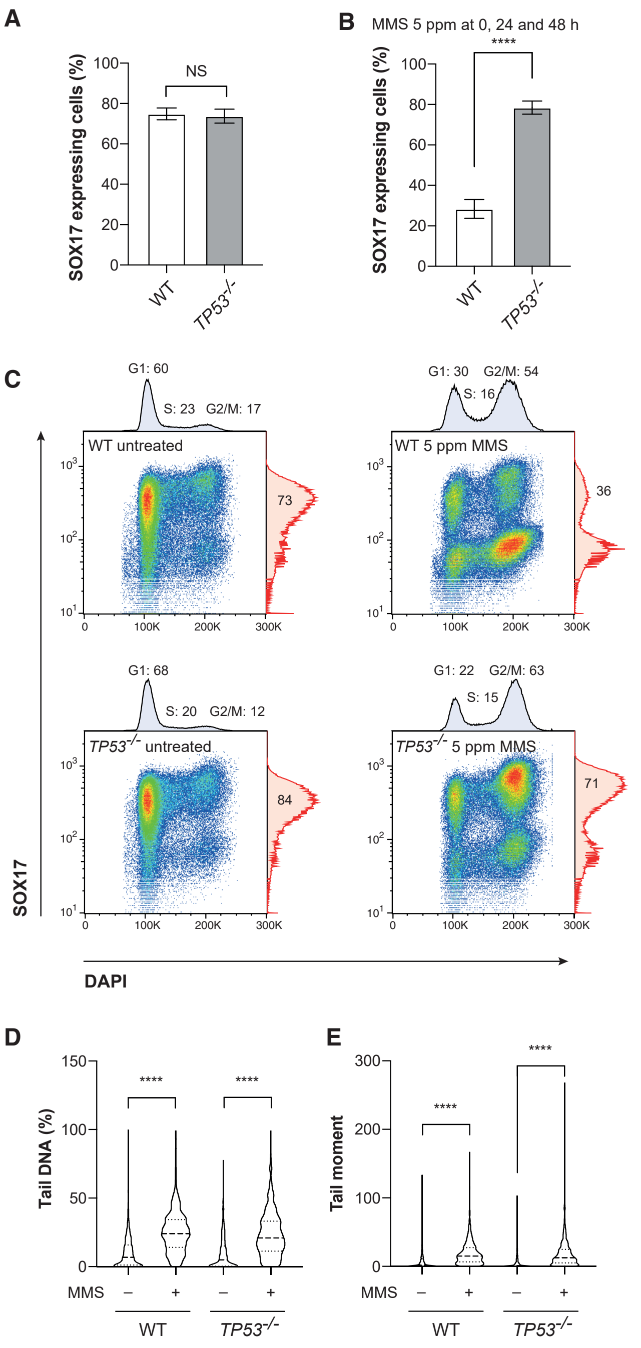

Figure 3. Loss of *TP53* Rescues DE Differentiation after DNA Damage

(A) The percentage of cells expressing SOX17 at 72 h of differentiation in the wild-type (WT) and $TP53^{-/-}$ cell line. No difference in the efficiency of differentiation was seen between the two cell lines using an unpaired t test ($p = 0.8126$, $n = 6$ independent experiments, mean ± SEM is plotted).

(B) WT and $TP53^{-/-}$ cell lines were treated with 5 ppm MMS at 0, 24, and 48 h into differentiation (when the medium was changed), and efficiency of treated cells was expressed as a percentage of untreated cells. Significance was calculated using an unpaired t test (****$p < 0.0001$, $n = 6$ independent experiments, mean ± SEM is plotted).

(C) WT and $TP53^{-/-}$ cells were differentiated either without treatment or with exposure to 5 ppm MMS at 0, 24, and 48 h. Cells were collected at 72 h and monitored for SOX17 expression and cell-cycle phase using DAPI. The SOX17-positive proportion of cells and the proportion of cells in each phase of the cell cycle are shown. Cell-cycle quantification was calculated using the Dean-Jett-Fox model on FlowJo.

(D) Alkaline comet analysis was performed on WT and $TP53^{-/-}$ differentiating cells. Cells were treated with or without 5 ppm MMS at 24 h and collected 2 h later for analysis ($n = 2$ independent experiments with >400 comets analyzed per condition; p values calculated with Kruskal-Wallis with Dunn's multiple comparisons test, ****$p < 0.0001$).

(E) As for (D) but showing tail moment, ****$p < 0.0001$.

Loss of p53 Rescues DE Differentiation after DNA Damage

To explore whether the DNA-damage-induced stabilization of p53 was linked to the failure to acquire markers of DE, we differentiated a $TP53^{-/-}$ BOBSC cell line (see Supplemental Experimental Procedures and Figure S3A) with and without exposure to MMS. The untreated $TP53^{-/-}$ and wild-type cells differentiated with the same efficiency (Figure 3A). However, $TP53^{-/-}$ cells differentiated with a much higher efficiency than the wild-type cell line when treated with MMS during differentiation (Figure 3B), while exhibiting a similar G2/M cell-cycle block (Figure 3C). It is noteworthy that although loss of p53 reduces the fraction of cells in G1 during MMS exposure, consistent with the known role of p53 in the G1/S checkpoint (Smith et al., 2020), the predominant accumulation of cells is in G2/M in both wild-type and, to a greater extent, $TP53^{-/-}$ cells (Figure 3C). This likely reflects both the chronic, low-dose DNA damage used in these experiments and the significant reliance on S-phase recombination pathways for tolerating the replication-stalling lesions created by MMS (Lundin et al., 2005). Disruption of *TP53* also rescued differentiation when MMS and UV were administered at 24 h only (Figures S3B and S3C). The restoration of efficient differentiation in MMS-treated $TP53^{-/-}$ cells was not explained by a change in the number of cell divisions (Figure S3D) and occurred despite similar levels of MMS-induced DNA damage detectable in alkaline comets, compared with wild-type cells (Figures 3D and 3E). Thus, the loss of p53 is sufficient to apparently rescue DE differentiation in the face of DNA damage.

To understand the nature of the p53-dependent changes in the DE transcriptional program induced by DNA damage, we performed time-resolved RNA sequencing during endoderm differentiation and in undifferentiated cells,

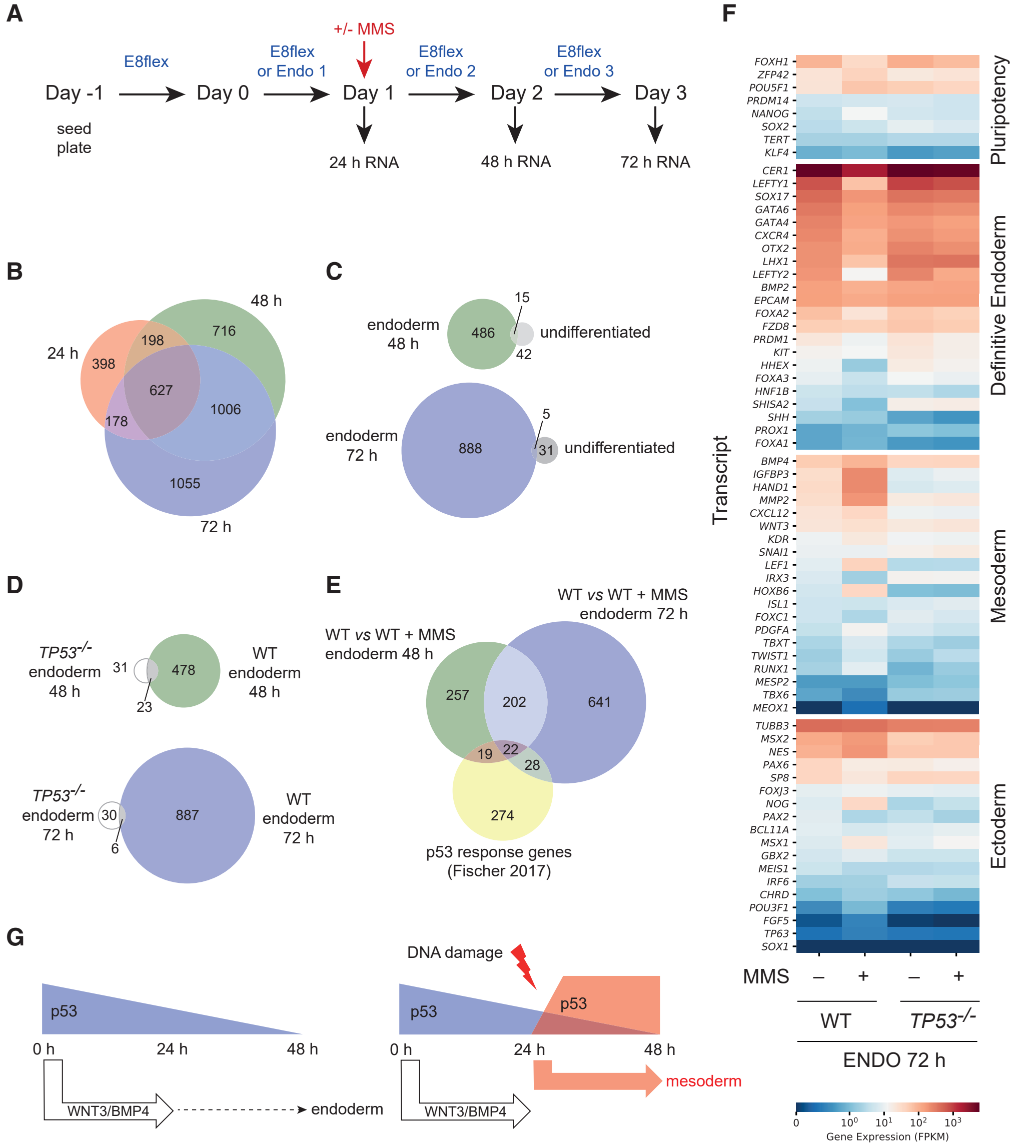

Figure 4. DNA-Damage-Induced Activation of p53 Enforces a Damage-Induced Transcriptional Program that Drives Differentiation toward Mesoderm

(A) Schematic to show the workflow of the experimental setup for RNA sequencing. On day −1 WT and *TP53*$^{-/-}$ cells were passaged, and on day 0 the experiment was initiated. Undifferentiated cells were cultured, in parallel to the differentiating cells, in Essential 8 Flex (E8flex) medium. RNA was extracted from both cell lines in the undifferentiated state and during differentiation every 24 h (days 1, 2, 3). In parallel, cells were treated with 5 ppm MMS at 24 h and RNA was extracted at days 2 and 3.

(B) Gene expression changes during differentiation: differentially expressed genes between untreated WT cells undergoing differentiation and undifferentiated cells collected at 24 h are shown. The overlap between the differences is shown at 24, 48, and 72 h of differentiation.

(C) Comparison of the differentially expressed genes after MMS treatment compared with untreated cells in the wild-type cell line: during differentiation (left) and undifferentiated cells (right). The top shows 48 h (24 h after treatment) and the bottom 72 h (48 h after treatment).

(D) Comparison of both WT and *TP53*$^{-/-}$ with and without MMS treatment during differentiation. The difference between treated and untreated cells at 48 h (top) and 72 h (bottom) is shown.

(E) A Venn diagram to show the overlap of the list of p53-response genes (Fischer, 2017) with those altered during differentiation after MMS treatment.

(F) Heatmap to show the expression of different lineage-specific markers and pluripotency markers at 72 h of differentiation in both WT and *TP53*$^{-/-}$ cell lines, with and without MMS treatment at 24 h.

(legend continued on next page)

with and without exposure to 5 ppm MMS at 24 h, in both wild-type and *TP53*$^{-/-}$ cells (Figure 4A).

DNA-Damage-Induced p53 Enforces a Transcriptional Program that Drives Differentiation toward Mesoderm

As expected, vast changes in gene expression accompanied endoderm differentiation (Figure 4B and Table S1). Treatment with MMS imposed significant additional changes on gene expression in the cells undergoing endoderm differentiation but had much less effect on undifferentiated cells (Figure 4C). The vast majority of the changes induced by MMS during differentiation were dependent on p53 (Figure 4D). Interestingly, while known p53 targets were transcriptionally upregulated 24 h after exposure to MMS in differentiating cells (Figure S4), only a small percentage of the genes affected are described as robust p53 targets (Figure 4E). This may reflect the strong bias in somatic cell p53 targets in the published literature (Fischer, 2017). Nonetheless, KEGG pathway analysis identifies p53 signaling as the most significantly affected pathway at this time (Table S2). Gene ontology (GO) analysis of the genes differentially expressed at 48 h into differentiation between wild-type treated and untreated cells revealed the most significant terms to be development, morphogenesis, and differentiation (Table S3). This suggests that MMS treatment was specifically affecting differentiation-related pathways. Inclusion of terms involving nervous system development also suggested that MMS treatment may alter lineage specification. At 72 h the significance of the developmental and morphogenesis terms was even more marked (Table S3). The number of genes significantly deregulated by MMS in the *TP53*$^{-/-}$ cell line was low (Figure 4D), and thus the GO analysis revealed no significant terms at 48 h, while those that reached significance at 72 h (Table S3) were involved in ion transport, which has previously been linked to p53 function (Mak et al., 2017).

To explore whether DNA damage does indeed redirect differentiation in a p53-dependent manner, we examined changes in expression in key pluripotency and lineage-specific markers (Saili et al., 2019) in each condition (Figure 4F). MMS-treated wild-type cells expressed lower levels of DE markers and higher levels of most mesoderm markers, along with small changes in ectoderm markers. This effect was almost exclusively dependent on p53, demonstrating that the DNA-damage-induced activation of p53 during a critical window of DE specification in hiPSCs alters the trajectory of differentiation.

DISCUSSION

Our observation that exposure to a low dose of a DNA-damaging agent during a critical window in the differentiation of hiPSCs to DE disturbs the efficiency and fidelity of the differentiation program is consistent with a recent study showing that overproduction of endogenous reactive oxygen species perturbs endoderm differentiation in hiPSCs, reducing *SOX17* expression and creating cells with tumorigenic potential in nude mice (Oka et al., 2020). Our study shows that the DNA-damage-induced redirection of differentiation is entirely dependent on the upregulation of p53. This p53-dependent response occurs without significant purging of damaged cells, suggesting that differentiating hiPSCs lack the “safety net” of an apoptotic response, highlighting the potential risk of damaged and transcriptionally perturbed cells remaining in the population.

Although *Trp53* is dispensable for broadly normal development in the mouse (Donehower et al., 1992), the *Trp53* family, which includes *Trp63* and *Trp73*, has been proposed to act redundantly to initiate mesendoderm differentiation by direct transcriptional regulation (Wang et al., 2017). Interestingly, neither *TP63* nor *TP73* was expressed (Table S4), suggesting that they were unlikely to be substituting for *TP53* in our system. Our observations suggest that the downregulation of p53 during DE differentiation may be necessary to allow the normal transcriptional program to proceed. This is consistent with previous work showing that while p53 is necessary for cells to initiate the EMT, attenuation of its levels by MDM2 is necessary to allow expression of the mesenchymal phenotype (Araki et al., 2010; Chang et al., 2011). Indeed, downregulation of p53 may be directly controlled by this transition, as the EMT factor TWIST1 is able to bind to p53, leading to MDM2-dependent degradation (Piccinin et al., 2012). We suggest that unscheduled stabilization of p53, caused by DNA damage during the EMT, results in a transcriptional perturbation driving differentiation away from DE (Figure 4G). Although the apical transcriptional targets of p53 upregulation in this context remain to be defined, we speculate that the effect could be, at least in part, mediated by extended WNT/BMP signaling (Gertow et al., 2013; Kempf et al., 2016; Lindsley et al., 2006).

It will be important to determine if the effect of p53 stabilization on differentiation after DNA damage that we observe in the lines studied here is generalizable to all human stem cells. However, if p53-dependent

(G) A model to suggest how DNA damage induces p53 stabilization and this leads to a change in gene expression in these cells, altering the outcome of differentiation. The upregulation of p53 in this context causes upregulation of mesoderm-lineage-specific genes, thus altering the trajectory of differentiation.

reprogramming of endoderm differentiation in response to low doses of DNA damage also occurs during human embryonic development, it may provide a mechanism by which damaged cells could be diverted away from forming germ cells, which are formed from the endodermal lineage. More extensive damage may well lead to increased apoptosis, as has been observed in irradiated mouse embryos (Heyer et al., 2000), and to a disordered embryo that would likely be lost before birth.

EXPERIMENTAL PROCEDURES

hiPSC and hESC Culture

Cells were cultured at 37°C, 5% CO_2 in Essential 8 or Essential 8 Flex medium on 6 well plates coated in Vitronectin-XF (STEMCELL Technologies). Cells were passaged 1:10 every 3–4 days depending on confluency. Cells were detached from the plates by a wash in 1 mL of 0.5 mM EDTA (Thermo Fisher Scientific) followed by incubation in fresh EDTA for 5 min before the EDTA was removed and the cells were blasted with medium to detach them. Cells were maintained as small clumps. Endoderm differentiation was driven as described (Yiangou et al., 2019) (Figure 1B, with full details in Supplemental Experimental Procedures).

MMS Treatment/UV-C Irradiation

MMS (Sigma) was freshly diluted 1000-fold in cell medium and vortexed before being added to cells at the final concentration. UV-C was delivered in a custom-built shuttered cabinet with a stabilized bulb output measured with a calibrated UV-C meter (UVP, Inc). Prior to irradiation, cell medium was replaced with 1 mL PBS.

Cell Division and Cell Death Assays

Cell division was monitored with a CellTrace Violet cell proliferation kit (Invitrogen). Cell death was determined using the FITC Annexin V apoptosis detection kit with 7-AAD (Biolegend). Full details of both procedures are provided in the Supplemental Experimental Procedures.

Flow Cytometry

For intracellular staining, *ca.* 1×10^6 cells were collected, having been detached from the plate with 1 mL Cell Dissociation Buffer (Thermo Fisher Scientific) for 10 min at 37°C. Following centrifugation in a 1.5 mL tube (1,500 × *g*, 4 min), the cell pellet was fixed in 200 μL 1% paraformaldehyde for 10 min at room temperature, repelleted, and resuspended in 200 μL of 90% PBS/10% DMSO and kept at −80°C until preparation for flow cytometry.

Fixed cells were thawed at room temperature for 10 min, pelleted (1,500 × *g*, 4 min), and resuspended in 200 μL 1× BD Perm/Wash buffer. Samples were divided in two to provide an IgG control, pelleted, and resuspended in 100 μL BD buffer. Cells were permeabilized and blocked (15 min, room temperature) and spun down for antibody staining: either primary followed by secondary or conjugated antibody staining. For antibodies used see Supplemental Experimental Procedures.

Protein Extraction, SDS-PAGE, and Western Blotting

Cells from a single well of a 6 well plate were harvested as described for flow cytometry and protein was extracted using RIPA buffer and standard methods. Full details and antibodies used are given in the Supplemental Experimental Procedures.

Alkaline Comet Assay

The R&D Systems comet assay kit was used according to the manufacturer's instructions. Further details are in the Supplemental Experimental Procedures. More than 100 comets were analyzed for each replicate of each condition. The CometScore 2.0 software (TriTek Corp.) was used to calculate the average tail moment.

RNA Extraction and Quantitative Reverse Transcription PCR

RNA extraction was performed using the RNeasy Qiagen RNA extraction kit and reverse transcribed with the Qiagen QuantiTect reverse transcription kit, using 800 ng RNA. qPCR was performed with SYBR Green Mastermix (Applied Biosystems) and a ViiA7 real-time qPCR system (Applied Biosystems). Full details are in the Supplemental Experimental Procedures.

Library Preparation for RNA Sequencing and RNA-Sequencing Data Analysis

RNA was extracted and libraries were prepared using standard methods (detailed in Supplemental Experimental Procedures). Raw sequencing data were trimmed for adaptor sequences with a minimum quality threshold of 30 using TrimGalore v.0.4.4 (https://github.com/FelixKrueger/TrimGalore). Trimmed reads were aligned to the human genome version GRCh38.87 using TopHat v.2.1.0 (Kim et al., 2013) and quantified per genomic region and differential expression was calculated using Cufflinks v.2.2.1 (Trapnell et al., 2013). Gene lists for Venn diagrams were created using the following cutoffs: fragments per kilobase of transcript per million mapped reads (FPKM) ≥ 1 for both samples, $\log_2$ fold change minimum of 1, and significantly different expression taken as calculated from CuffDiff (Trapnell et al., 2013). The Python library Matplotlib (Hunter, 2007) was used to produce Venn diagrams from lists of differentially expressed genes, and Seaborn (https://doi.org/10.5281/zenodo.3629446) was used for generating heatmaps, plotting the mean FPKM per triplicate for each condition.

Data and Code Availability

The RNA-sequencing data have been deposited in GEO (https://www.ncbi.nlm.nih.gov/geo/) with accession no. GSE146225.

SUPPLEMENTAL INFORMATION

Supplemental Information can be found online at https://doi.org/10.1016/j.stemcr.2020.08.003.

AUTHOR CONTRIBUTIONS

C.B.E. performed all experiments and analysis and formed the project. F.J.A. generated the scripts for analysis of RNA-sequencing data and the Venn diagrams and heatmaps; A.C. performed the

initial RNA-sequencing alignments and read counts. R.G. and L.V. hosted C.B.E. in the early stages of the project and provided critical guidance on the differentiation system. J.E.S. was responsible for conceptualization and supervision of the project. The paper was written by C.B.E. and J.E.S.

ACKNOWLEDGMENTS

We thank Maria Daly and her team in the LMB flow cytometry facility for assistance, the Wellcome Trust Sanger Institute and COMSIG consortium for providing the wild-type and $TP53^{-/-}$ BOBSC cells, Benedicte Recolin for assistance with cell culture, and lab colleagues for comments on the manuscript. The work was supported by a central grant to the Laboratory of Molecular Biology from the UK Medical Research Council (U105178808).

Received: April 17, 2020
Revised: August 7, 2020
Accepted: August 7, 2020
Published: September 3, 2020

REFERENCES

Andersson-Rolf, A., Mustata, R.C., Merenda, A., Kim, J., Perera, S., Grego, T., Andrews, K., Tremble, K., Silva, J.C., Fink, J., et al. (2017). One-step generation of conditional and reversible gene knockouts. Nat. Methods *14*, 287–289.

Araki, S., Eitel, J.A., Batuello, C.N., Bijangi-Vishehsaraei, K., Xie, X.J., Danielpour, D., Pollok, K.E., Boothman, D.A., and Mayo, L.D. (2010). TGF-beta1-induced expression of human Mdm2 correlates with late-stage metastatic breast cancer. J. Clin. Invest. *120*, 290–302.

Armstrong, J.F., Kaufman, M.H., Harrison, D.J., and Clarke, A.R. (1995). High-frequency developmental abnormalities in p53-deficient mice. Curr. Biol. *5*, 931–936.

Arnold, S.J., Hofmann, U.K., Bikoff, E.K., and Robertson, E.J. (2008). Pivotal roles for eomesodermin during axis formation, epithelium-to-mesenchyme transition and endoderm specification in the mouse. Development *135*, 501–511.

Banin, S., Moyal, L., Shieh, S., Taya, Y., Anderson, C.W., Chessa, L., Smorodinsky, N.I., Prives, C., Reiss, Y., Shiloh, Y., and Ziv, Y. (1998). Enhanced phosphorylation of p53 by ATM in response to DNA damage. Science *281*, 1674–1677.

Beranek, D.T. (1990). Distribution of methyl and ethyl adducts following alkylation with monofunctional alkylating agents. Mutat. Res. *231*, 11–30.

Canman, C.E., Lim, D.S., Cimprich, K.A., Taya, Y., Tamai, K., Sakaguchi, K., Appella, E., Kastan, M.B., and Siliciano, J.D. (1998). Activation of the ATM kinase by ionizing radiation and phosphorylation of p53. Science *281*, 1677–1679.

Chang, C.J., Chao, C.H., Xia, W., Yang, J.Y., Xiong, Y., Li, C.W., Yu, W.H., Rehman, S.K., Hsu, J.L., Lee, H.H., et al. (2011). p53 regulates epithelial-mesenchymal transition and stem cell properties through modulating miRNAs. Nat. Cell Biol. *13*, 317–323.

Cordenonsi, M., Dupont, S., Maretto, S., Insinga, A., Imbriano, C., and Piccolo, S. (2003). Links between tumor suppressors: p53 is required for TGF-beta gene responses by cooperating with Smads. Cell *113*, 301–314.

Donehower, L.A., Harvey, M., Slagle, B.L., McArthur, M.J., Montgomery, C.A., Butel, J.S., and Bradley, A. (1992). Mice deficient for p53 are developmentally normal but susceptible to spontaneous tumours. Nature *356*, 215–221.

Dufort, D., Schwartz, L., Harpal, K., and Rossant, J. (1998). The transcription factor HNF3beta is required in visceral endoderm for normal primitive streak morphogenesis. Development *125*, 3015–3025.

Fischer, M. (2017). Census and evaluation of p53 target genes. Oncogene *36*, 3943–3956.

Gertow, K., Hirst, C.E., Yu, Q.C., Ng, E.S., Pereira, L.A., Davis, R.P., Stanley, E.G., and Elefanty, A.G. (2013). WNT3A promotes hematopoietic or mesenchymal differentiation from hESCs depending on the time of exposure. Stem Cell Reports *1*, 53–65.

Godley, L.A., Kopp, J.B., Eckhaus, M., Paglino, J.J., Owens, J., and Varmus, H.E. (1996). Wild-type p53 transgenic mice exhibit altered differentiation of the ureteric bud and possess small kidneys. Genes Dev. *10*, 836–850.

Heyer, B.S., MacAuley, A., Behrendtsen, O., and Werb, Z. (2000). Hypersensitivity to DNA damage leads to increased apoptosis during early mouse development. Genes Dev. *14*, 2072–2084.

Hunter, J.D. (2007). Matplotlib: a 2D graphics environment. Comput. Sci. Eng. *9*, 90–95.

Jones, S.N., Roe, A.E., Donehower, L.A., and Bradley, A. (1995). Rescue of embryonic lethality in Mdm2-deficient mice by absence of p53. Nature *378*, 206–208.

Kanai-Azuma, M., Kanai, Y., Gad, J.M., Tajima, Y., Taya, C., Kurohmaru, M., Sanai, Y., Yonekawa, H., Yazaki, K., Tam, P.P., and Hayashi, Y. (2002). Depletion of definitive gut endoderm in Sox17-null mutant mice. Development *129*, 2367–2379.

Kempf, H., Olmer, R., Haase, A., Franke, A., Bolesani, E., Schwanke, K., Robles-Diaz, D., Coffee, M., Göhring, G., Dräger, G., et al. (2016). Bulk cell density and Wnt/TGFbeta signalling regulate mesendodermal patterning of human pluripotent stem cells. Nat. Commun. *7*, 13602.

Kim, D., Pertea, G., Trapnell, C., Pimentel, H., Kelley, R., and Salzberg, S.L. (2013). TopHat2: accurate alignment of transcriptomes in the presence of insertions, deletions and gene fusions. Genome Biol. *14*, R36.

Lee, K.H., Li, M., Michalowski, A.M., Zhang, X., Liao, H., Chen, L., Xu, Y., Wu, X., and Huang, J. (2010). A genomewide study identifies the Wnt signaling pathway as a major target of p53 in murine embryonic stem cells. Proc. Natl. Acad. Sci. U S A *107*, 69–74.

Lin, T., Chao, C., Saito, S., Mazur, S.J., Murphy, M.E., Appella, E., and Xu, Y. (2005). p53 induces differentiation of mouse embryonic stem cells by suppressing Nanog expression. Nat. Cell Biol. *7*, 165–171.

Lindsley, R.C., Gill, J.G., Kyba, M., Murphy, T.L., and Murphy, K.M. (2006). Canonical Wnt signaling is required for development of embryonic stem cell-derived mesoderm. Development *133*, 3787–3796.

Lundin, C., North, M., Erixon, K., Walters, K., Jenssen, D., Goldman, A.S., and Helleday, T. (2005). Methyl methanesulfonate

(MMS) produces heat-labile DNA damage but no detectable in vivo DNA double-strand breaks. Nucleic Acids Res. *33*, 3799–3811.

Mak, T.W., Hauck, L., Grothe, D., and Billia, F. (2017). p53 regulates the cardiac transcriptome. Proc. Natl. Acad. Sci. U S A *114*, 2331–2336.

Michael, D., and Oren, M. (2003). The p53-Mdm2 module and the ubiquitin system. Semin. Cancer Biol. *13*, 49–58.

Montes de Oca Luna, R., Wagner, D.S., and Lozano, G. (1995). Rescue of early embryonic lethality in mdm2-deficient mice by deletion of p53. Nature *378*, 203–206.

Oka, S., Hayashi, M., Taguchi, K., Hidaka, M., Tsuzuki, T., and Sekiguchi, M. (2020). ROS control in human iPS cells reveals early events in spontaneous carcinogenesis. Carcinogenesis *41*, 36–43.

Parant, J., Chavez-Reyes, A., Little, N.A., Yan, W., Reinke, V., Jochemsen, A.G., and Lozano, G. (2001). Rescue of embryonic lethality in Mdm4-null mice by loss of Trp53 suggests a nonoverlapping pathway with MDM2 to regulate p53. Nat. Genet. *29*, 92–95.

Piccinin, S., Tonin, E., Sessa, S., Demontis, S., Rossi, S., Pecciarini, L., Zanatta, L., Pivetta, F., Grizzo, A., Sonego, M., et al. (2012). A "twist box" code of p53 inactivation: twist box: p53 interaction promotes p53 degradation. Cancer Cell *22*, 404–415.

Rogakou, E.P., Pilch, D.R., Orr, A.H., Ivanova, V.S., and Bonner, W.M. (1998). DNA double-stranded breaks induce histone H2AX phosphorylation on serine 139. J. Biol. Chem. *273*, 5858–5868.

Sah, V.P., Attardi, L.D., Mulligan, G.J., Williams, B.O., Bronson, R.T., and Jacks, T. (1995). A subset of p53-deficient embryos exhibit exencephaly. Nat. Genet. *10*, 175–180.

Saili, K.S., Antonijevic, T., Zurlinden, T.J., Shah, I., Deisenroth, C., and Knudsen, T.B. (2019). Molecular characterization of a toxicological tipping point during human stem cell differentiation. Reprod. Toxicol. *91*, 1–13.

Schmid, P., Lorenz, A., Hameister, H., and Montenarh, M. (1991). Expression of p53 during mouse embryogenesis. Development *113*, 857–865.

Shiloh, Y., and Ziv, Y. (2013). The ATM protein kinase: regulating the cellular response to genotoxic stress, and more. Nat. Rev. Mol. Cell Biol. *14*, 197–210.

Singh, I., Ozturk, N., Cordero, J., Mehta, A., Hasan, D., Cosentino, C., Sebastian, C., Krüger, M., Looso, M., Carraro, G., et al. (2015). High mobility group protein-mediated transcription requires DNA damage marker γ-H2AX. Cell Res. *25*, 837–850.

Smith, H.L., Southgate, H., Tweddle, D.A., and Curtin, N.J. (2020). DNA damage checkpoint kinases in cancer. Expert Rev. Mol. Med. *22*, e2.

Teo, A.K., Arnold, S.J., Trotter, M.W., Brown, S., Ang, L.T., Chng, Z., Robertson, E.J., Dunn, N.R., and Vallier, L. (2011). Pluripotency factors regulate definitive endoderm specification through eomesodermin. Genes Dev. *25*, 238–250.

Thomson, J.A., Itskovitz-Eldor, J., Shapiro, S.S., Waknitz, M.A., Swiergiel, J.J., Marshall, V.S., and Jones, J.M. (1998). Embryonic stem cell lines derived from human blastocysts. Science *282*, 1145–1147.

Trapnell, C., Hendrickson, D.G., Sauvageau, M., Goff, L., Rinn, J.L., and Pachter, L. (2013). Differential analysis of gene regulation at transcript resolution with RNA-seq. Nat. Biotechnol. *31*, 46–53.

Vallier, L., Touboul, T., Brown, S., Cho, C., Bilican, B., Alexander, M., Cedervall, J., Chandran, S., Ahrlund-Richter, L., Weber, A., and Pedersen, R.A. (2009). Signaling pathways controlling pluripotency and early cell fate decisions of human induced pluripotent stem cells. Stem Cells *27*, 2655–2666.

Wallingford, J.B., Seufert, D.W., Virta, V.C., and Vize, P.D. (1997). p53 activity is essential for normal development in Xenopus. Curr. Biol. *7*, 747–757.

Wang, Q., Zou, Y., Nowotschin, S., Kim, S.Y., Li, Q.V., Soh, C.L., Su, J., Zhang, C., Shu, W., Xi, Q., et al. (2017). The p53 family coordinates Wnt and nodal inputs in mesendodermal differentiation of embryonic stem cells. Cell Stem Cell *20*, 70–86.

Williams, A.B., and Schumacher, B. (2016). p53 in the DNA-damage-repair process. Cold Spring Harb. Perspect. Med. *6*, a026070.

Yiangou, L., Grandy, R.A., Morell, C.M., Tomaz, R.A., Osnato, A., Kadiwala, J., Muraro, D., Garcia-Bernardo, J., Nakanoh, S., Bernard, W.G., et al. (2019). Method to synchronize cell cycle of human pluripotent stem cells without affecting their fundamental characteristics. Stem Cell Reports *12*, 165–179.

Yusa, K., Rashid, S.T., Strick-Marchand, H., Varela, I., Liu, P.Q., Paschon, D.E., Miranda, E., Ordóñez, A., Hannan, N.R., Rouhani, F.J., et al. (2011). Targeted gene correction of α1-antitrypsin deficiency in induced pluripotent stem cells. Nature *478*, 391–394.

Stem Cell Reports, Volume *15*

Supplemental Information

A p53-Dependent Checkpoint Induced upon DNA Damage Alters Cell Fate during hiPSC Differentiation

Cara B. Eldridge, Finian J. Allen, Alastair Crisp, Rodrigo A. Grandy, Ludovic Vallier, and Julian E. Sale

Supplemental Information for:

A p53-dependent checkpoint induced upon DNA damage alters cell fate during hiPSC differentiation

Cara B. Eldridge [1], Finian J. Allen [2], Alastair Crisp [1], Rodrigo A. Grandy [3], Ludovic Vallier [3,4,5] and Julian E. Sale [1*]

[1] MRC Laboratory of Molecular Biology, Francis Crick Avenue, Cambridge, CB2 0QH, UK
[2] Department of Chemistry, University of Cambridge, Cambridge, CB2 1EW, UK
[3] Wellcome–MRC Cambridge Stem Cell Institute, Anne McLaren Laboratory, University of Cambridge, Cambridge CB2 0SZ, UK
[4] Department of Surgery, University of Cambridge, Cambridge CB2 0QQ, UK
[5] Wellcome Sanger Institute, Wellcome Genome Campus, Hinxton CB10 1SA, UK

[*] to whom correspondence should be addressed: jes@mrc-lmb.cam.ac.uk

Figure S1 (Related to Figure 1).

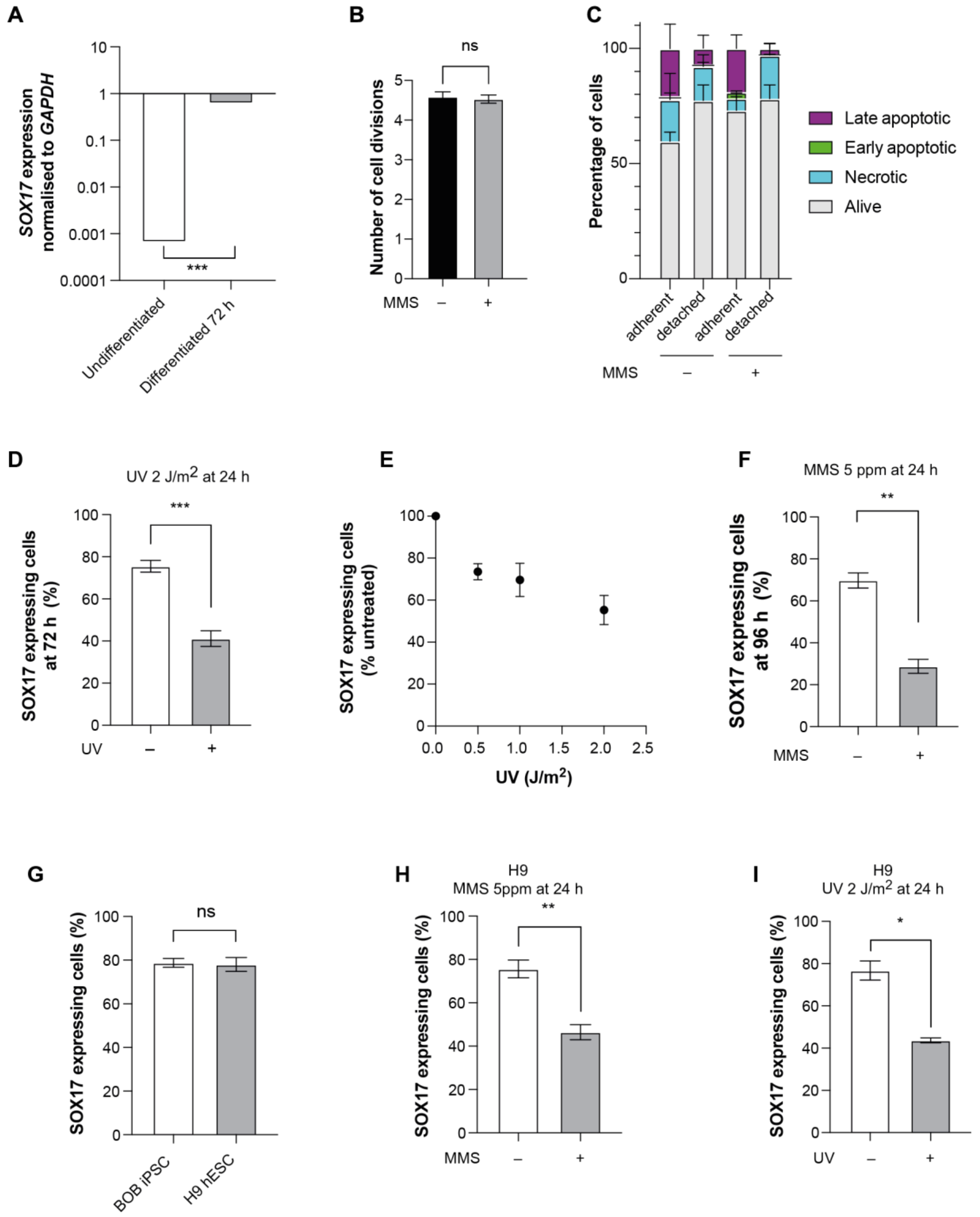

(A) *SOX17* expression calculated using RT-qPCR of undifferentiated and 72 hour differentiating cells normalised to *GAPDH*. (Mean ± SD, n = 2 independent experiments, $p < 0.0004$ using a paired t test.

(B) CellTrace™ Violet was used to analyse the number of cell divisions during differentiation with and without 5 ppm MMS treatment. Cells were incubated with the dye for 20 minutes prior to the set-up of differentiation and then Time 0 cells were collected to analyse the initial fluorescence. Differentiation was then set up in with cells either treated with MMS at 24 hours or untreated, all samples were collected at 72 hours and monitored by flow cytometry. The number of cell cycles was calculated by comparing to the reading at Time 0 (see STAR methods) (n = 4 independent experiments, mean ± SEM, no significant difference was detected between any samples using paired t test).

(C) Viability assay monitoring Annexin V and 7-AAD to measure the proportion of cells undergoing necrosis or apoptosis. Cells were treated with 5 ppm MMS at 24 hours of differentiation and viability was monitored at 48 hours (24 hours later). Adherent cells were those remaining attached to the culture dish whereas detached cells were those that lost adherence. (n = 4 independent experiments, mean ± SD).
(D) Wild-type cells were irradiated with 2 J/m^2 UV-C at 24 hours, or left untreated, and cells were collected at 72 hours to monitor with proportion of SOX17 positive cells (n = 5, p = 0 .0007, paired t-test).
(E) UV-C irradiation dose response in wild-type BOBSC cells. Cells were irradiated at 24 hours and SOX17 expression was monitored at 72 hours (n = 2 independent experiments per time point, mean ± SEM).
(F) Wild-type cells were differentiated with or without 5ppm MMS treatment at 24 hours and differentiation was continued to 96 hours (24 hours longer than usual) to see if cells could catch up with SOX17 expression (n = 4 independent experiments, p = 0.0062 using paired t test).
(G) Wild-type BOBSC hiPS cells were differentiated in parallel with H9 hES cells and the percentage of cells expressing SOX17 at 72 hours was calculated (n = 6 independent experiments, p = 0.8589, unpaired t test mean ± SEM is plotted).
(H) H9 cells were differentiated with or without 5 ppm MMS treatment at 24 hours and the expression of SOX17 at 72 hours was monitored (n = 4 independent experiments, p = 0.0023 using a paired t-test mean ± SEM is plotted).
(I) H9 cells were differentiated with or without 2 J/m^2 UV-C irradiation at 24 hours (n = 3 independent experiments, p = 0.0222 using paired t test mean ± SEM is plotted.)

Figure S2 (Related to Figure 2).

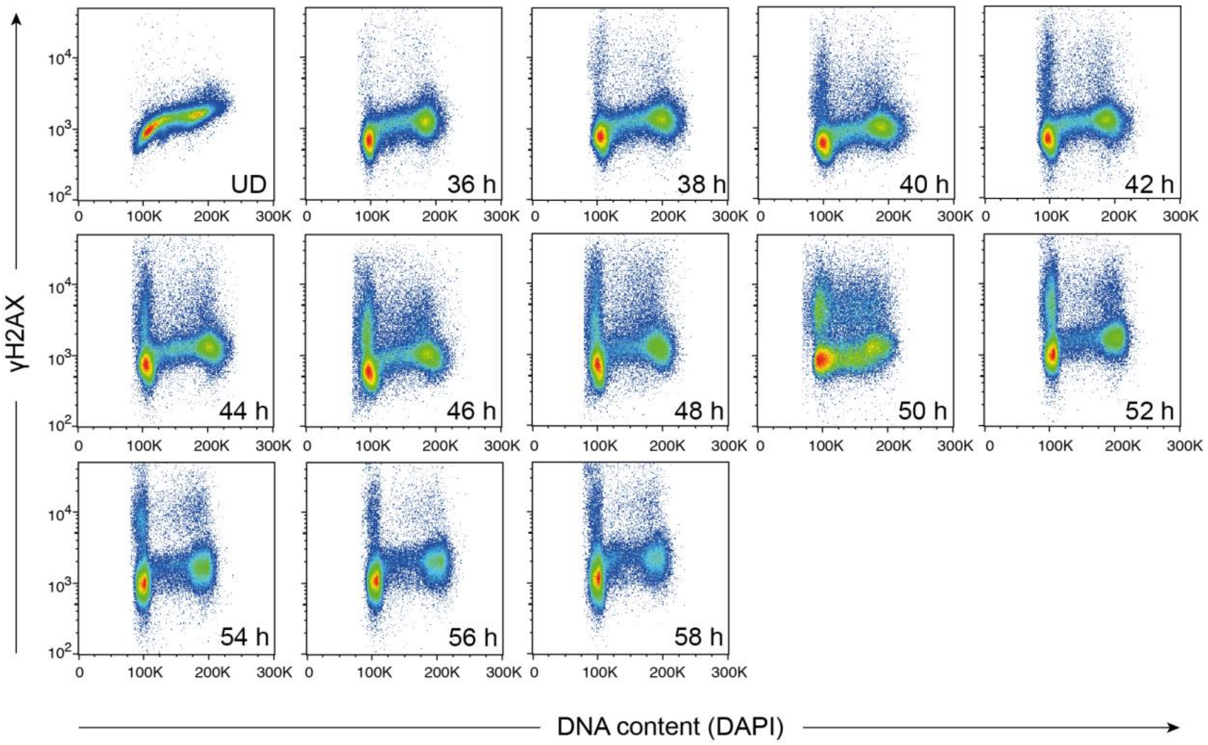

Flow cytometry analysis of the phosphorylation of H2AX during untreated differentiation of wild-type cells. Undifferentiated cells and cells at different times during differentiation were collected and fixed, and then the level of H2AX phosphorylation was monitored with DAPI staining to monitor the position in the cell cycle.

Figure S3 (Related to Figure 3).

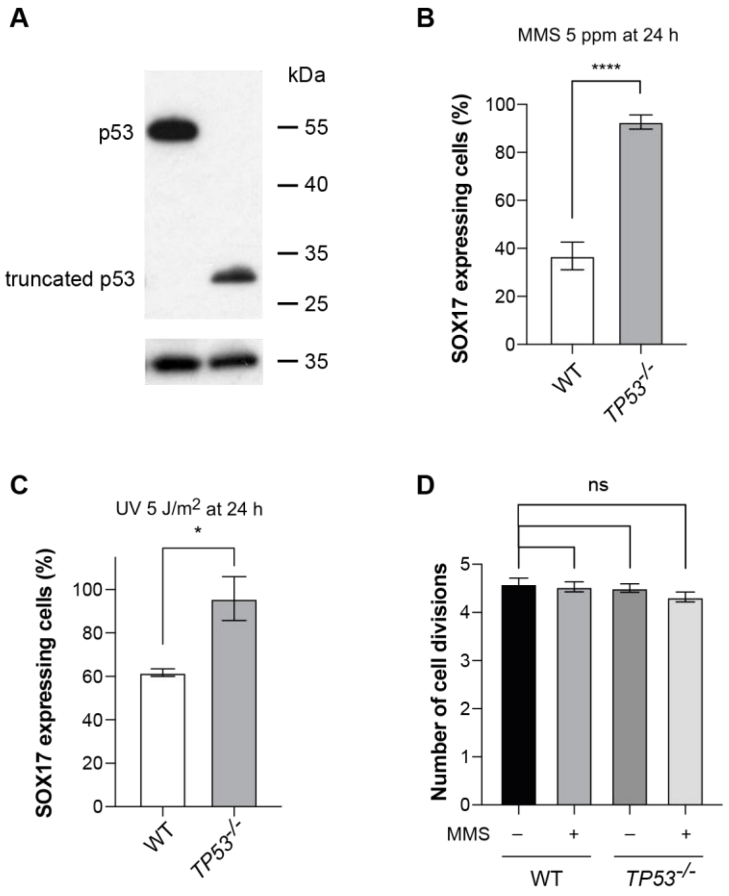

(A) Western blot to show the level of total p53 in the wild-type BOBSC and *TP53*$^{-/-}$ cell lines 5 h after 20 J/m^2 UV-C irradiation to induce p53 stabilisation, β-ACTIN was used as a loading control. The p53 DO-1 antibody binds to amino acids 11-25 of human p53 and therefore detects the N-terminus.

(B) Wild-type and *TP53*$^{-/-}$ cells were differentiated with or without 5 ppm MMS treatment at 24 hours and cells were collected for permeabilised flow cytometry at 72 hours. SOX17 expression after MMS is expressed as a percentage of the untreated level of expression. Wild-type is shown on the left and *TP53*$^{-/-}$ is shown on the right (n = 6 independent experiments, p < 0.0001 using unpaired t test, mean ± SEM is plotted).

(C) As for B. but using 5 J/m^2 UV-C irradiation at 24 hours (n = 4 independent experiments, p = 0.0161 using unpaired t test, mean ± SEM is plotted).

(D) S1C but with *TP53*$^{-/-}$ cells added (n = 4, independent experiments; no significance was detected between any samples using one-way ANOVA).

Figure S4 (Related to Figure 4).

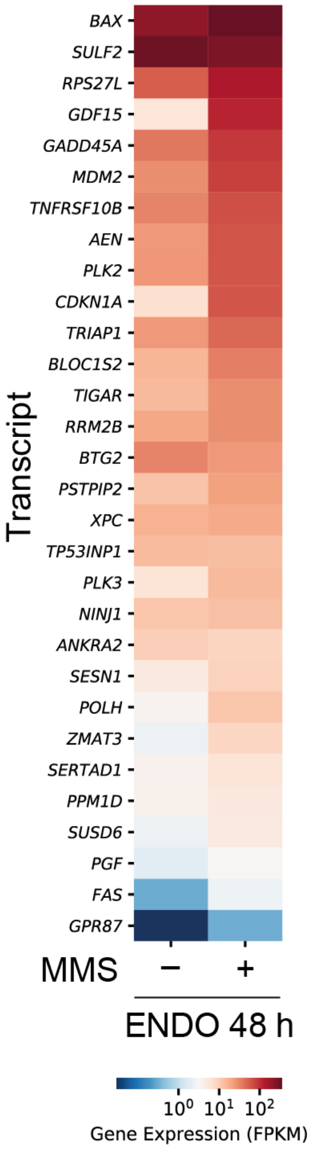

A heatmap to show the expression levels of p53-response genes, expressed as mean FPKM values, in the wild-type cell line at 48 hours, with and without MMS treatment at 24 hours.

Supplemental Tables

Supplemental Table 1 (Related to Figure 4).
Differentially expressed genes.
See separate Excel spreadsheet.

Supplemental Table 2 (Related to Figure 4).

Term ID	Description	Adjusted p-value	Count in selected genes	Count in all genes
KEGG:04115	p53 signaling pathway	0.000694	11	72
KEGG:04371	Apelin signaling pathway	0.0358	12	137
KEGG:05200	Pathways in cancer	0.0542	27	526
KEGG:05205	Proteoglycans in cancer	0.0542	14	199
KEGG:04151	PI3K-Akt signaling pathway	0.0595	20	353
KEGG:05216	Thyroid cancer	0.104	5	37
KEGG:05218	Melanoma	0.104	7	72
KEGG:04510	Focal adhesion	0.19	11	199
KEGG:04060	Cytokine-cytokine receptor interaction	0.19	15	292
KEGG:05225	Hepatocellular carcinoma	0.19	10	167

KEGG pathway analysis using genes differentially expressed in wild-type untreated compared to MMS treated cells Kyoto Encyclopedia of Genes and Genomes **(**KEGG) analysis of wild-type 48 h differentiating cells treated with and without MMS. Benjamini-Hochberg multiple testing correction was used to calculate probabilities. Only the top two pathways were significant.

Supplemental Table 3 (Related to Figure 4).

Wild type				
48 hours				
Term ID	Description	Adjusted p-value	Count in selected genes	Count in all genes
GO:0048856	anatomical structure development	6.30×10^{-19}	218	5793
GO:0032502	developmental process	8.68×10^{-19}	227	6212
GO:0007275	multicellular organism development	1.04×10^{-16}	200	5321
GO:0048731	system development	1.17×10^{-15}	183	4760
GO:0009653	anatomical structure morphogenesis	1.87×10^{-15}	123	2598
GO:0032501	multicellular organismal process	2.05×10^{-15}	245	7414
GO:0007399	nervous system development	3.60×10^{-15}	113	2299
GO:0030154	cell differentiation	6.24×10^{-15}	163	4088
GO:0023051	regulation of signaling	9.58×10^{-15}	146	3482
GO:0010646	regulation of cell communication	9.58×10^{-15}	145	3447
72 hours				
GO:0009653	anatomical structure morphogenesis	2.31×10^{-32}	236	2598
GO:0048856	anatomical structure development	5.53×10^{-32}	395	5793

GO:0007275	multicellular organism development	$5.53x10^{-32}$	373	5321
GO:0032502	developmental process	$1.56x10^{-29}$	407	6212
GO:0009887	animal organ morphogenesis	$1.79x10^{-29}$	126	969
GO:0048731	system development	$1.42x10^{-26}$	332	4760
GO:0048513	animal organ development	$4.95x10^{-25}$	262	3428
GO:0032501	multicellular organismal process	$9.50x10^{-25}$	445	7414
GO:0048646	anatomical structure formation involved in morphogenesis	$8.86x10^{-23}$	126	1144
GO:0009888	tissue development	$3.20x10^{-22}$	173	1926
TP53$^{-/-}$				
72 hours				
GO:0008324	cation transmembrane transporter activity	0.0119	7	664
GO:0046873	metal ion transmembrane transporter activity	0.0119	6	457
GO:0022803	passive transmembrane transporter activity	0.0119	6	470
GO:0015267	channel activity	0.0119	6	469
GO:0005261	cation channel activity	0.0119	5	320
GO:0015075	ion transmembrane transporter activity	0.0292	7	897
GO:0022890	inorganic cation transmembrane transporter activity	0.0292	6	605
GO:0015077	monovalent inorganic cation transmembrane transporter activity	0.0292	5	392
GO:0005215	transporter activity	0.0301	8	1251
GO:0022842	narrow pore channel activity	0.0312	2	19

GO analysis of differentially expressed genes in wild-type and *TP53$^{-/-}$* cells with and without MMS treatment, at 48 and 72 hours of differentiation Gene ontology (GO) analysis of the untreated cell line compared to the MMS treated cell line at 48 hours and 72 hours. PANTHER was used to perform enrichment analysis and Benjamini-Hochberg multiple testing correction was used to calculate probabilities. No significant terms were seen for *TP53$^{-/-}$* at 48 hours using $p < 0.05$.

Supplemental Table 4 (Related to Figure 4).

	TP53	***TP63***	***TP73***
Undifferentiated	107.7761	0.069823	0.765466
24 h differentiated	85.87637	0.119782	0.594662
48 h differentiated	84.11267	0.065392	0.324827
72 h differentiated	101.423	0.118819	0.70977

Expression levels of the *TP53* family during differentiation. Gene expression of members of the *TP53* family expressed as average FPKM (n = 3) in wild-type untreated cells during differentiation. The undifferentiated sample is the 24 h undifferentiated sample. An FPKM > 1 was used as the threshold for detection of expression.

Supplemental Experimental Procedures

hiPS and hES cells

BOBSC cells (Andersson-Rolf et al., 2017), a derivative of cA1ATD cells (Yusa et al., 2011), were obtained from the Sanger Centre. p53-deficient BOBSC lines were generated by the COMSIG (Causes of Mutational SIGnatures) project, funded by the Wellcome Trust, using a CRISPR/Cas9 strategy in which one allele was disrupted by gene targeting and the other by error-prone repair.

Cell line	Sanger HTGT plate code	Barcode	Targeting
WT cA1ATD (BOBSC)		BOBSC-T6/8_B1	n/a
TP53 KO	HUFP0007_3_A_H04	1095765195	Exon 6

The WA09 (H9) hES cells were obtained from WiCell via Dr. Madeline Lancaster, MRC LMB, Cambridge. They were cultured as for the BOBSC cells.

Endoderm Differentiation protocol

Cells were passaged 1:8 onto Vitronectin-XF (StemCell Technologies) coated six well plates one day prior to setting up endoderm differentiation. On day one of differentiation, cell medium was changed to CDM-PVA supplemented with 100 ng/mL Activin A (R&D), 80 ng/mL FGF2 (R&D), 10 ng/mL BMP4 (R&D), 10 µM PI3K inhibitor LY294002 (Promega) and 3 µM GSK3i CHIR99021 (Tocris). On day two, cell medium was replaced with CDM-PVA supplemented with Activin A, FGF2, BMP4 and LY294002. CDM-PVA consisted of 50% Ham's F-12 (Gibco) and 50% IMDM (Gibco) supplemented with 1 g/L PVA (Sigma), 1 mM concentrated Lipids (Life technologies), 0.5 mM Thioglycerol (Sigma), 15 µg/mL Transferrin (Roche) and 7 µg/mL Insulin (Roche). The PVA solution was first made up in ultrapure water by heating to 90˚C with stirring, continuing overnight while cooling to ensure the powder was dissolved. On day three, the medium was replaced with RPMI+ supplemented with 100 ng/mL Activin A and 80 ng/mL FGF2. RPMI was supplemented with 1 mM NEM-NEEA (Gibco) and B27 supplement (Yiangou et al., 2019).

Cell division assay

Cell divisions were monitored with CellTrace™ Violet Cell Proliferation Kit (Invitrogen™). Cells were loaded with 5 µM CellTrace™ reagent in PBS for 20 min at 37˚C. The cells were washed three times with warm medium and incubated until analysis. Cells should lose half of the fluorescence at each cell division. Therefore, comparing the fluorescence of the cells collected at the start and end of differentiation, the number of cell divisions can be quantified using the equation below.

$$N = \frac{\log\left(\frac{Co}{Ct}\right)}{\log 2}$$

Cell death assay

Cell death was determined using the FITC Annexin V Apoptosis Detection Kit with 7-AAD (Biolegend®). Both adherent and non-adherent cell fractions were collected and pelleted at 300 *g* for 4 min. Cells were washed twice in Cell Staining Buffer and then resuspended in 100 µL Annexin V Binding Buffer per well of a six well plate. The cells were transferred to a flow cytometry tube and 5 µL FITC Annexin V and 5 µL 7-AAD was added. The cells were vortexed and incubated in the dark at room temperature for 15 min). 400 µL Annexin V Binding Buffer was added to each tube and the cells were analysed using flow cytometry.

Antibody staining of permeabilised cells

For monitoring γH2AX, permeabilised cells were resuspended in 100 µL anti-γH2AX antibody (1/500) (Merck Millipore 05-636) (4°C, overnight) or IgG control without antibody. Cells were spun down (1500 *g*, 4 min), washed in BD buffer twice and resuspended in secondary antibody (1/200) (Invitrogen Alexa Fluor®) (1 h, RT, dark). Cells were spun down and washed, as above, and resuspended in 400 µL PBS/BSA 0.5% with 1 µg DAPI per sample and protected from light until analysis. For staining SOX17 and EOMES, directly conjugated antibodies were used, as below. Cells were resuspended in 100 µL BD buffer containing 4 µL conjugated antibody, or appropriate isotype control, per reaction and incubated in the dark (30 min, RT). Cells were washed

once in BD buffer, spun down (1500 *g*, 4 min) and resuspended in 400 μL PBS/BSA 0.5% with 1 μg DAPI per sample for analysis.

Protein	Conjugated Primary Antibody
SOX17	BD Pharmingen™ 562205 Alexa Fluor® 488 Ms
EOMES	Invitrogen 50-4877-42 eBioscience™ eFluor® 660 Ms

All flow cytometry was performed on a BD LSRFortessa™, which is equipped with 405 nm, 488 nm, 561 nm and 640 nm lasers with analysis using FlowJo®, LLC, initially gating out debris using FSC/SSC and then gating for single cells using DAPI staining V-450A/V-450H.

Protein extraction, SDS-PAGE and western blotting

Cells from a single well of a six well plate were harvested as described for flow cytometry. Following two washes in 500 µL PBS, cells were resuspended in 100 µL per 500,000 cells RIPA buffer (Cell Signalling) containing Benzonase® nuclease (1/1000) and 1X Halt™ Protease and Phosphatase inhibitor cocktail (Thermo Scientific). Eppendorfs containing cells and buffer were rotated slowly (4˚C, 1 h) and then transferred to a cold centrifuge (16,000 *g*, 4˚C, 30 min). The supernatant (protein) was transferred to a clean tube, being careful not to disturb the DNA pellet at the bottom. 5X SDS-PAGE sample buffer (Sigma) was added to 1X and the protein was boiled (95˚C, 5 min). Protein was stored at -20˚C until use. Protein was thawed on ice and then was run on a NuPAGE® Bis-Tris 4-12% precast gel (Thermo Fisher) in 1X MOPS buffer (50 mM MOPS, 50 mM Tris, 0.1% SDS, 1 mM EDTA, pH 7.7) at 120 V for 2 h. Semi-dry transfers to nitrocellulose were performed using the iBlot® 2 system (Thermo Fisher) using iBlot® 2 transfer stacks at 25 V for 7 min. The membrane was put into deionised water, cut to size and stained with Ponceau S (15 s, RT, rotating) to check sample loading and transfer efficiency. The membrane was washed twice and blocked in 5% milk (Marvel) in 1X TBST (150 mM NaCl, Tris HCl pH7.4 10 mM, 0.1% tween) (1 h, RT, rotating).

Primary antibodies were added as stated in the table below. Membranes were incubated with primary antibody overnight, rotating at 4˚C. The membrane was washed three times for 5-15 minutes while rotating at room temperature with 1X TBST. The membrane was then blotted with secondary antibody (1 h, RT, rotating). All secondary antibodies were HRP-conjugated (Dako P0447-9) used at 1/5000 dilution. The secondary antibody was washed as for the primary antibody and signal revealed with Millipore Luminata Crescendo reagent.

Antibodies

Protein epitope	Dilution	Catalogue no.	Manufacturer	Species
β-ACTIN	1:20,000	Ab8227	Abcam	Rabbit
pCHK1 (Ser-345) (133D3)	1:1000	113D3	CST	Rabbit
CHK1	1:1000	Ab40866	Abcam	Rabbit
pCHK2 (Thr-68)	1:1000	2661	CST	Rabbit
CHK2	1:50,000	Ab109413	Abcam	Rabbit
γH2AX (Ser-139) JBW301	1:5000	05-636	Merck Millipore	Mouse
H2AX total	1:500	SC-517336	Santa Cruz	Mouse
p-p53 (Ser15)	1:1000	9284	CST	Rabbit
p53 (DO-1)	1:1000	Ab1101	Abcam	Mouse
PC10 (PCNA)	1:10,000	SC-56	Santa Cruz	Mouse
MDM2	1:1000	Ab259265	Abcam	Rabbit
pRPA32 (Ser-33)	1:1000	A300-246A	Bethyl	Rabbit
RPA32	1:1000	Ab2175	Abcam	Mouse

Alkaline comet assay

The R&D systems comet assay kit was used according to the manufacturer's instructions. Briefly, cells were washed in ice cold DPBS and resuspended at $1x10^5$ cells/ mL in ice cold DPBS. 50 μL cells were added to 500 μL warm low melting point agarose and mixed before pipetting onto two wells of the comet slide. Slides were left at 4˚C in the dark for one hour and then kept in cold lysis buffer at 4°C for six days. Slides were immersed in alkaline unwinding solution (200 mM NaOH, 1 mM EDTA) (20 min, 4˚C) prior to electrophoresis. Cells were electrophoresed in the same solution (32 V, 300 mA, 30 min). Slides were washed twice in ultrapure water followed by 70% ethanol for 20 minutes. Slides were dried and stained with SybrGold before analysis on the microscope.

RNA extraction and quantitative reverse transcription PCR (RT-qPCR)

Medium was aspirated from adherent cells and replaced with 600 µL RLT buffer containing β-mercaptoethanol. Cells were transferred to sterile Eppendorf tubes and stored at -80˚C or processed further immediately. Extraction was performed according to the manufacturer's instructions, but without the initial spin to remove debris. RNA was eluted into 30 µL nuclease free water and 1 µL was analysed on the nanodrop to check RNA concentration and for contamination with DNA or protein. RNA was stored at -80˚C. RNA was reverse transcribed to DNA using Qiagen QuantiTect® reverse transcription kit using 800 ng RNA. The initial gDNA 'wipeout' step was performed (42˚C, 2 min) and the reverse transcription (42˚C, 25 min). cDNA was stored at -20˚C for up to 48 hours before qPCR was performed.

The qPCR master mix was made up of 50% SYBR® Green Mastermix (Applied Biosystems), 10 µM Fwd and 10 µM Rv primer and 10% diluted cDNA in DEPC. Reactions were set up on a 96 or 384-well optical plate in 20 µL volumes in triplicate and sealed with optical film. The plate was spun down (3220 *g*, 1 min) and run on a ViiA7™ real-time qPCR system (Applied Biosystems) for 45 cycles (hold stage: 50˚C, 2 min and 95˚C, 10 min and PCR stage: 95˚C, 15 s and 60˚C, 1 min).

**Transcript**	**Fwd Primer: 5'-3'**	**Rv Primer: 5'-3'**
GAPDH	TCACCAGGGCTGCTTTTAACT	GACGGTGCCATGGAATTTGC
SOX17	CGCACGGAATTTGAACAG TA	GGATCAGGGACCTGTCACAC

Library preparation for RNA sequencing

RNA was extracted and quantified as above before running on an Agilent RNA Pico 6000 chip using an Agilent 2100 Bioanalyzer. RNA with a RIN score above 7 was used to generate RNA libraries. 750 ng of RNA was diluted into 50 µL DEPC water and kept on ice. RNA libraries were prepared using the NEBNext UltraII RNA library preparation kit (New England Biolabs E7770) and PolyA tail enrichment with eight PCR cycles and the NEBNext Oligos 1-24 and then stored at -20˚C until analysis. One µg of each constructed library was run on an Agilent 2100 Bioanalyzer High Sensitivity DNA chip and the electrogram was analysed for shape of the graph and the average size of each library (bp): the library was checked for primer (~80 bp) and adapter (~128 bp) contamination and any libraries with high primer/adapter content were re-purified. The libraries were next quantified using the KAPA Library Quantification Kit for Illumina® (Roche). Libraries were serially diluted to 1/100000 and 1/1000000 in 10 mM Tris-HCl pH 8.0 and quantified in triplicate as stated in the protocol using the Rox-low buffer for the ViiA7 qPCR system (Thermo Fisher Scientific Melt-curves for the libraries were also analysed to assess the quality of the libraries. Average C_Ts were transferred to the KAPA quantification excel spreadsheet with standard values and the concentration of each library was calculated. After quantification, libraries created with different NEBNext Oligos were pooled to a total concentration of 20 nM in 10 mM Tris-HCl pH 8.0. The pooled library was run on a High Sensitivity DNA chip, as above, and the average size of the library calculated before sending to sequencing. 20 µL of pooled library was sequenced on a HiSeq4000 machine with single end reads.
